# CRL4^DCAF13^ E3 ubiquitin ligase targets MeCP2 for degradation to prevent DNA hypermethylation and ensure normal transcription in growing oocytes

**DOI:** 10.1007/s00018-024-05185-4

**Published:** 2024-04-05

**Authors:** Peipei Ren, Xiaomei Tong, Junjian Li, Huifang Jiang, Siya Liu, Xiang Li, Mengru Lai, Weijie Yang, Yan Rong, Yingyi Zhang, Jiamin Jin, Yerong Ma, Weiwei Pan, Heng-Yu Fan, Songying Zhang, Yin-Li Zhang

**Affiliations:** 1 Assisted Reproduction Unit, Department of Obstetrics and Gynecology, Sir Run Run Shaw Hospital, School of Medicine, Zhejiang University, Hangzhou, 310016 China; 2 Key Laboratory of Reproductive Dysfunction Management of Zhejiang Province, Hangzhou, China; 3 College of Life and Environmental Science, Wenzhou University, Wenzhou, China; 4 Department of Cell Biology, College of Medicine, Jiaxing University, Jiaxing, 314001 China; 5 Life Sciences Institute, Zhejiang University, Hangzhou, 310058 China

**Keywords:** Oocyte aging, Ovarian aging, Transcriptional dysregulation, WGBS, E3 ligase, Protein ubiquitination

## Abstract

The DNA methylation is gradually acquired during oogenesis, a process sustained by successful follicle development. However, the functional roles of methyl-CpG-binding protein 2 (MeCP2), an epigenetic regulator displaying specifical binding with methylated DNA, remains unknown in oogenesis. In this study, we found MeCP2 protein was highly expressed in primordial and primary follicle, but was almost undetectable in secondary follicles. However, in aged ovary, MeCP2 protein is significantly increased in both oocyte and granulosa cells. Overexpression of MeCP2 in growing oocyte caused transcription dysregulation, DNA hypermethylation, and genome instability, ultimately leading to follicle growth arrest and apoptosis. MeCP2 is targeted by DCAF13, a substrate recognition adaptor of the Cullin 4-RING (CRL4) E3 ligase, and polyubiquitinated for degradation in both cells and oocytes. *Dcaf13*-null oocyte exhibited an accumulation of MeCP2 protein, and the partial rescue of follicle growth arrest induced by *Dcaf13* deletion was observed following *MeCP2* knockdown. The RNA-seq results revealed that large amounts of genes were regulated by the DCAF13-MeCP2 axis in growing oocytes. Our study demonstrated that CRL4^DCAF13^ E3 ubiquitin ligase targets MeCP2 for degradation to ensure normal DNA methylome and transcription in growing oocytes. Moreover, in aged ovarian follicles, deceased DCAF13 and DDB1 protein were observed, indicating a potential novel mechanism that regulates ovary aging.

**Supplementary Information:**

The online version contains supplementary material available at 10.1007/s00018-024-05185-4.

## Introduction

Oogenesis occurs within a follicle that encompasses several cell types, such as granulosa cells (GCs), theca cells, and oocyte. Folliculogenesis commences with a pool of quiescent primordial follicles and concludes with the ovulation of a viable mature oocyte or the cessation of follicular death by atresia [1]. Following the transitional process from the primordial follicle to the primary follicle, also known as follicle activation [2, 3], sustained follicle growth is dependent on bidirectional communication between the oocyte and GCs. Emerging evidence has been shown that the oocyte governs the developmental fate of the follicle by secreting some factors, such as GDF9 and BMP15 [4, 5]. Abnormalities in early folliculogenesis cause ovarian aging, premature ovarian failure (POF), and female sterility [6, 7].

Generally, DNA methylation repressed chromatin state and transcription activity. Notably, de novo DNA methylation during oogenesis displays unique features [8]. Oocytes in primordial follicles have low level of DNA methylation, and the onset of DNA methylation coincides with the transition of mouse oocytes from primary to secondary follicles when they attain a diameter of about 50 µm [9]. The DNA methyltransferases (DNMT3A and co-factor DNMT3L) de novo methylate genome-wide DNAs at both CpG and non-CpG loci, a process that is nearly completed in fully-grown germinal vesicle (GV) oocyte [10, 11]. Low-input sequencing analysis revealed that DNA methylation is mainly enriched in gene bodies with active transcription, while un-transcribed regions are poorly methylated. Moreover, DNA methylation is dependent on transcription and is associated with the permissive chromatin state [12–14].

DNA methylation generally correlates with chromatin-associated gene silencing [15]. While de novo DNA methylation is known to depend on active transcription in oogenesis [16], the mechanism by which gradually increased DNA methylation in the oocyte marginally affects transcription remains a mystery. MeCP2, also known as methyl-CpG-binding protein 2, serve as a mediator between DNA methylation and gene transcription [17, 18]. MeCP2 was initially characterized as a transcriptional repressor based on the presence of its transcriptional repressor domain [19, 20]. Recent studies have demonstrated that MeCP2 serves as a multifunctional factor, functioning as a transcription repressor, activator, and modulator of chromatin structure [21, 22]. The MeCP2 protein is prevalent in the brain, and its deficiency or gain-of-function causes severe neurodevelopmental disorders: Rett syndrome (RTT) and duplication syndrome (MDS), respectively [23]. As a result, MeCP2 research has mostly focused on the brain. MeCP2 is widely expressed in various tissues, exhibiting cell type and differentiation stage-specific distribution [24]. Several recent investigations in various organ anomalies, such as pneumonia, heart failure, skeletal muscle fibrosis, and precocious puberty with hyperandrogenism in male [25–28]. However, the role of MeCP2 in the ovary is yet unclear.

The Cullin4-RING (CRL4) E3 ligase is a multiprotein complex that specifically recognizes substrates for ubiquitin addition following catalyzation by the E1 activation enzyme and E2 conjugation enzyme [29]. The CRL4 E3 ligase is composed of Cullin4A or 4B (CUL4A/B) serving as a scaffold, DNA damage-binding protein-1 (DDB1) acting as an adapter that connects with the receptor, and ROC1/2 functioning as a RING finger protein to bind with the ubiquitin-charged E2-formed catalytic core [29]. A class of proteins known as DDB1 and CUL4 associated factors (DCAFs) are responsible for substrate recognition and determining substrate specificity [30]. In addition to DNA damage, cell cycle regulation, and virus infection, the CRL4 E3 ligase is engaged in epigenetic regulation via polyubiquitination or mono-ubiquitination of the substrates, such as WDR5 and TET1-3 [31–34]. Our previous studies have demonstrated that oocyte-specific deletion of *Ddb1* induced premature ovarian failure, highlighting the vital role of CRL4 E3 ligase in oocyte survival and follicle growth [34–36]. The CRL4^DCAF^ [1] E3 ligase activates TET1-3 catalytic activities by mono-ubiquitination in oocytes [34]. Deletion of *Ddb1* in oocytes induces DNA hypermethylation and follicle atresia [34]. DCAF13 functions as a substrate recognizer in the CRL4 E3 ligase complex, and *Dcaf13* deletion in the oocyte caused premature ovarian failure [6, 37]. This means that the CRL4^DCAF13^ E3 ligase complex plays important roles in oogenesis and follicle growth.

In this study, we discovered that the MeCP2 protein was abundant in primordial follicles, but dramatically decreased during active follicle growth. MeCP2 protein was significantly higher in aged ovarian follicles in comparison to young ones. Thus, we investigated the function and protein degradation mechanism of MeCP2 in the ovary. This study demonstrated a novel mechanism that the CRL4^DCAF13^ E3 ligase targets MeCP2 for degradation, thereby preventing DNA hypermethylation and preserving normal transcription. Meanwhile, the aberration of the DCAF13-MeCP2 axis offers insight into ovarian aging.

## Materials and methods

### Animals

All animal experimental procedures were carried out in accordance with the Animal Research Committee guidelines of Zhejiang University. Wild-type ICR mice were obtained from the Shanghai SLAC Laboratory Animal Co., Ltd, China. All transgenic mouse strains were generated and maintained on a C57BL/6 background. *Dcaf13*^*flox/flox*^*; Gdf9-Cre* mice were previously generated and were a gift from H.Y. Fan [6]. *Ddx4-Rfp* mice were previously reported [38]. All animals were housed in a specific pathogen-free (SPF) environment with a 12 h light/12 h dark cycle and fixed temperature (21–23 ℃).

### Ovary, follicle and oocyte collection

The ovaries were collected from female ICR mice at day 1, 5, 10, and 14. The 2-month-old and 11-month-old *Ddx4-Rfp* female mice were sacrificed for ovaries collection and subsequent frozen sections. Ovaries from 14-day-old mice were punctured using sterile needles to obtain growing oocytes and secondary follicles. Mice at 23 days of age were injected with 5 IU of pregnant mare serum gonadotropin (PMSG, San-sheng Phamathetical) and were humanely euthanized 48 h later. The fully-grown GV oocytes were harvested and cultured in M2 medium (M7167, Sigma-Aldrich) covered with mineral oil at 37 °C in a 5% CO_2_ atmosphere.

### Follicle culture

Secondary follicles containing two layers of granulosa cells were isolated from the ovaries of 14-day-old female ICR mice in M2 medium. The culture medium utilized for follicle culture is α-MEM (Gibco), supplemented with 0.23 mM pyruvate (S8636, Sigma-Aldrich), 0.1 IU/ml FSH (Gonal-f, Merck Serono), 50ug/ml Vitamin C (Sigma), 5%FBS (Gibco), 1% penicillin and streptomycin (Gibco), 1 × Insulin-Transferrin-Selenium (ITS-G, Gibco). Follicles culture at 37 °C and 5% CO_2_ in air, change half medium every other day.

### Plasmids

The FLAG- or Myc-tagged plasmids of DDB1, DCAF13, and CUL4A were previously described [35]. The FLAG-H2A.X were previously reported and a gift from H.Y. Fan [39]. Full-length human *MeCP2* cDNA was obtained from the human Ultimate ORF clone library. After sequencing, FLAG-MeCP2 and mCherry-MeCP2 expression plasmids were generated using LR clonase (11791019, Thermo Fisher Scientific), according to the manufacturer’s instructions.

### In vitro transcription and microinjection

The FLAG-tagged *MeCP2* plasmids and pcDNA3.1 + -mCherry plasmids were linearized using *BglII* and *SmaI* restriction enzymes, respectively. The mCherry*-MeCP2* plasmids were digested by *EcoRI*. With the linearized plasmids as template, the 5'-capped mRNAs were in vitro transcribed using Sp6 or T7 mMESSAGE mMACHINE Kits (AM1340, AM1344, Invitrogen, Carlsbad, CA, USA), followed by poly(A) tail addition using a Poly (A) Tailing Kit (AM1350, Invitrogen) according to the manufacturer’s instructions. Then, the DNA templates were digested by Turbo DNase, and the synthesized mRNAs were recovered by adding lithium chloride and purified with 70% ethanol. The cRNAs were dissolved in RNase-free water. The cRNAs (250 ng/ul) were microinjected into denuded growing oocytes or oocytes in secondary follicles using a Narishige micromanipulator.

### TUNEL assay for detection of DNase I sensitivity

Oocytes overexpressing FLAG or FLAG-MeCP2 were collected and pre-extracted immediately in ice-cold solution (50 mM NaCl, 3 mM MgCl_2_, 0.5% Triton X-100, and 300 mM sucrose in 25 mM 4-(2-hydroxyethyl)-1-piperazineethanesulfonic acid (HEPES), pH 7.4) for 5 min. The oocytes were incubated with different concentrations of DNase I for 5 min at 37 °C in the same buffer without Triton X-100 and fixed for 10 min in 2% PFA/PBS at room temperature. TUNEL assay was performed using Click-iT TUNEL Alexa Fluor Imaging Assay (Life Technologies, C10245) according to the manufacturer’s instructions.

### Cell culture, transfection and drug treatment

HeLa cells were cultured in Dulbecco's modified Eagle medium (DMEM) supplemented with 10% FBS (Gibco), 100 IU/ml penicillin, and 100 mg/ml streptomycin at 37 °C with 5% CO_2_. Plasmids and siRNAs were transfected into HeLa cells using Lipofectamine 2000 (11,668,019, Thermo Fisher Scientific) and Lipofectamine RNAiMAX (13,778,150, Thermo Fisher Scientific), respectively. The detailed siRNAs’ sequences are available in Supplementary Table 1.

HeLa cells were treated with 10 µM MG132 (HY-13259, MCE) or 10 µM MLN4924 (B1036, APExBio) for 0 h, 6 h, 12 h and 24 h. Hela cells transfected with indicated plasmids were treated with 10 µM cycloheximide (CHX, HY-13259, MCE) for specific durations.

### Estradiol levels detection

Estradiol levels were determined with a solid-phase, ligand-labeled, electrochemiluminescent enzyme-linked immunoassay using a Cobasw4000 automated random access immunoluminescence analyzer (Roche, Switzerland).

### Immunofluorescence analysis

The fresh ovaries were fixed overnight in 4% paraformaldehyde at 4 °C, followed by dehydration in 10% and 30% sucrose solutions. Subsequently, they were embedded in OCT compounds (Tissue-Tek, SAKURA) for sectioning. The follicles, oocytes, and cells were fixed with 4% paraformaldehyde at room temperature for 30 min. The oocytes were penetrated with 0.1% Triton X-100 in PBS at room temperature for 20 min before blocking with 1% BSA in PBS. The follicles and sections were simultaneously penetrated and blocked with blocking buffer (containing 5%BSA 0.3% Triton X-100) at room temperature for 1 h. Then, samples were incubated with primary antibodies (1:200) diluted in blocking solution at room temperature for 2 h or overnight at 4 °C. After three washes with PBS, the Alexa Fluor 488- or 568-conjugated goat anti-rabbit (A11001, A11036, Invitrogen, 1:300) or the Alexa Fluor 488- or 568-conjugated donkey anti-mouse (A21202, A10037, Invitrogen, 1:300) secondary antibodies were incubated with or without Alexa Fluor 647-conjugated phalloidin (A22287, Invitrogen, 1:200) for 1 h at room temperature. Then, 1 µg/ml DAPI (236,276, Roche, Basel, Switzerland) were used to co-stained the nuclei. After several times dense washing, the sections or samples were mounted, and the signals were captured using a laser scanning confocal microscope (LSM800, Carl Zeiss, Jena, Germany). The primary antibodies were listed in Supplementary Table 2.

### EU incorporation assay

Oocytes injected with mRNAs were cultured in M2 medium with 1 mM 5-ethynyl uridine (EU) for 1 h. Fixation, permeabilization, and staining were performed according to the manufacturer’s protocol of Click-iT RNA Alexa Fluor 488 Imaging kit (C10329, Thermo Fisher Scientific).

### Histological analysis

Ovary samples embedded in paraffin were sectioned (5 μm thick) for subsequent hematoxylin and eosin (HE) staining and immunohistochemistry (IHC). For IHC, sections were deparaffinized and rehydrated. Primary antibodies were applied at suitable dilutions at room temperature for 1 h, and samples were then incubated with biotinylated secondary antibodies for 30 min. The sections were then stained with Vectastain ABC and DAB peroxidase substrate kits (Vector Laboratories). The images were taken with a microscope system (Carl Zeiss). The diameters of oocytes and follicles were analyzed using Image J.

### Co-immunoprecipitation

Following a 48-h transfection, the cells were lysed using lysis buffer (containing 50 mM Tris–HCl, pH 7.5, 150 mM NaCl, 10% glycerol, and 0.5% NP-40, with the addition of freshly prepared protease and phosphatase inhibitors). After centrifugation at 12,000 g for 10 min, the supernatant was subjected to immunoprecipitation with anti-FLAG M2 magnetic beads (M8823, Sigma-Aldrich) or anti-c-Myc affinity agarose gels (E6654, Sigma-Aldrich). After incubation at 4 °C for 4 h, beads were washed three times with lysis buffer. The 4 × Laemmli sample buffer (1610747, Bio-Rad) was added to the beads, and the eluates were used for western blot analysis.

### In vivo ubiquitin assay

To detect protein ubiquitination status, cells were lysed in SDS denaturing buffer (20 mM Tris, pH 7.4, 50 mM NaCl, 0.5% NP-40, 0.5% sodium deoxycholate, 0.5% SDS, and 1 mM EDTA; protein inhibitors were added prior to use) after HA-Ub co-transfection with indicated plasmids for 48 h. The lysates were then boiled at 95 °C and immediately frozen at -80 °C. Then, cell lysates were thawed and diluted ten times with dilution buffer (50 mM Tris–HCl pH 7.4, 150 mM NaCl, 1 mM EDTA, 0.5% sodium deoxycholate, 1% NP-40, protein inhibitors were added before use). The samples were then incubated with anti-FLAG M2 magnetic beads (M8823, Sigma-Aldrich) for 2 h at 4 °C, beads then were washed three times with lysis buffer. The immunoprecipitation eluates were analyzed by western blotting using an anti-HA antibody.

### Western Blot

Fresh ovaries were lysed in RIPA buffer (P0013B, Beyotime) at 4 °C for 20 min after crushing, and the supernatant was harvested after centrifugation. Then, the 4 × Laemmli sample buffer containing β-mercaptoethanol was added. Oocyte samples or HeLa cells were lysed directly in 1 × Laemmli sample buffer. After sample boiling at 95 °C for 5 min, SDS–PAGE electrophoresis and immunoblots were performed following procedures as previously reported [40]. The antibodies used are listed in Supplementary Table 2. The quantification of protein levels was performed using Image J software.

### RNA extraction and qRT-PCR

Total RNAs were extracted from oocytes (n = 20) or granulosa cells using the RNeasy Mini Kit (74,104, Qiagen) according to the manufacturer’s instructions. The mRNAs were converted to first-strand cDNA using HiScript II Reverse Transcriptase (R201-01, Vazyme Scientific). The genes of interest were amplified using the CFX96 Real-Time System (Bio-Rad) and SYBR Green mix (R201-01, Vazyme Scientific). The mRNA levels were determined using the comparative Ct method, with the expression of mouse *Gapdh* or *Actb* serving as an internal control. The primers were designed based on the cDNA sequence of genes from the NCBI database and were detailed in Supplementary Table 3.

### Oocyte RNA sequencing (RNA-seq)

Oocytes (n = 5 per sample) were placed directly into lysis buffer (containing 0.2% Triton X-100, 10 mM dNTPs, 10 μM oligo-dT primer and RNase inhibitor) and subjected to cDNA synthesis according to the Smart-seq2 protocol described previously [6]. The cDNA libraries were prepared using KAPA HyperPlus Library Preparation Kit (KK8510, Roche). The quality of the cDNA libraries was assessed using a Bioanalyzer instrument (Agilent). The samples were subjected to sequencing by Illumina HiSeq 2500 for 125-bp paired-end sequencing with at least 4 million reads per single cell sample.

### RNA-seq data analysis

The RNA-seq data were processed with the standard procedure as previously reported [6]. In brief, the quality of the RNA-seq data was checked by FastQC (v0.11.8). Then, the raw reads were preprocessed using Trimmomatic (v0.35) to eliminate adapters, and the clean reads were mapped to the mouse reference genome (mm10) using TopHat (version 2.0.9). The gene counts were calculated using HTSeq (v0.6.1p1). The gene expression levels were quantified using normalized reads per kilobase per million mapped reads (RPKM). Differential expression genes were determined by Cufflinks (version 2.1.1) and R (version 3.5.1), with the significance threshold set at |log2 (fold change) |> 1 and *P* value < 0.05. Scatter plots were created using the ggplot2 packages in R, and heatmaps were plotted using pheatmap packages in R. Gene Ontology (GO) term analysis was conducted through the DAVID online (https://david-d.ncifcrf.gov/) and the Gene Set Enrichment Analysis (GSEA) was conducted using GSEA software (version 2.2.2). The Venn diagrams were drawn based on the results obtained from the Evolutionary Genomics online tool (http://bioinformatics.psb.ugent.be/webtools/Venn/).

### Library preparation for low-input whole-genome bisulfite sequencing (WGBS)

The genomic DNA of growing oocytes was extracted, and low-input WGBS libraries were prepared by E-GENE Co. Ltd, as previously descibed [41]. In brief, 100 oocytes or embryo nucleus were seeded into lysis buffer using a mouth pipette in each sample. Genomic DNA extraction was performed using the QIAamp DNA Micro Kit (Qiagen), and DNA quantification was carried out using the Quant-iT dsDNA HS Assay Kit with Invitrogen Qubit fluorometer. Then, a ZYMO EZ DNA Methylation-Gold Kit was utilized to convert unmethylated cytosine into uracil according to the instructions by the manufacturer. The purified DNA was subjected to post bisulfite extension with 0.4 mM dNTPs, 0.4 μM oligo 1 (5′-Biotin-CTACACGACGCTCTTCCGATCTNN NNNNNNN-5′) and 1 × Blue Buffer. After incubation at 65 °C for 3 min and 4 °C pause, Klenow Fragment (3′-5′ exo-) were added, and the samples were then incubated at 4 °C for 5 min, + 1 °C/15 s to 37 °C, 37 °C for 30 min. After the first-strand synthesis, 40U exonuclease I (NEB) was added and incubated for 1 h at 37 °C followed by DNA purification and amplified DNA capturing using streptavidin beads. The second-strand synthesis was carried out with 0.8 mM dNTPs and 4 μM oligo 2 (5′-TGCTGAACCGCTCTTCCGAT CTNNNNNNNNN-3′). The libraries were generated by PCR amplification using KAPA HiFi HotStart DNA Polymerase (KAPA Biosystems), following by analysis using an Agilent Bioanalyzer (Agilent Technologies) and real time PCR. Finally, the libraries were sequenced by Illumina Nova 6000 by E-GENE Co., Ltd.

### Raw data filtration, sequence alignment and methylation level

All bisulfite sequencing reads were first trimmed to eliminate low-quality sequences (q < 20) and adapters using Trimmomatic v0.38 [42]. The parameter settings used were “SLIDINGWINDOW:5:15 HEADCROP:3 AVGQUAL:15 LEADING:5 TRAILING:5 MINLEN:80”. The clean data were mapped to the *mm10* reference genome using BSMAP v2.90 [43] in paired-end alignment mode with the following parameters “-v 0.08 -g 1 -n 1 -p 48”. Finally, BatMeth2 [44] was employed to eliminate potential PCR duplicates and to extract the cytosine methylation ratio from the BSMAP mapping results. Methylation ratios were extracted from BSMAP output (SAM) using a Python script (methratio.py) that is included in the BSMAP package. In brief, the methylation level was calculated based on the methylated cytosine (mC) percentage in the whole genome as site methylation level = 100 × (number of sequences with methylated cytosines (mC)/total number of valid sequences). The global average methylation levels were visualized using a ViolinPlot in the R package. The average methylation levels of different gene elements were calculated by genome gff file and visualized by barplot using R package.

### DMR analysis

To obtain reliable differentially methylated regions (DMRs), methylKit [45] was used to identify a sliding-window approach with 1000-bp window with a step size of 500-bp. Subsequently, a t test was utilized to identify significant DMRs based on the following criteria: CpG number > 5, methylation difference threshold percentage (methdiff) > 10, q value of < 0.05, and the length of DMR over 50 bp. After that, differentially methylated genes (DMGs) were identified using bedtools v2.30.0 [46]. The DMGs and DEGs were intersected with online Venn Diagram-drawing tools available at https://bioinformatics.psb.ugent.be/webtools/Venn/. These overlapped genes were subjected to GO enrichment analysis by Allenricher (v1.0). Finally, IGV_2.12.3 was utilized for visualization of the methylation levels of DMGs [47]. Methylation levels of different genomic gene, CpG Island, and TE regions were acquired utilizing BatMeth2 and the resulting graphs were visualized using the ggplot2 packages in R software v4.1.0.

### Image acquisition and quantification

The Nikon Ts2R microscope was utilized for the acquisition of bright-field images of follicles. The Image J software was utilized for measuring follicle diameter. For the quantification of oocyte immunofluorescence (IF) signal intensity, fluorescent images in different groups were captured using an LSM800 laser scanning confocal microscope (Carl Zeiss) under identical parameters and subsequently analyzed using ImageJ software.

### Statistical analysis

All collected growing oocytes or secondary follicles from wild-type mice were randomized into control and MeCP2 overexpression group for microinjection. Then, the oocytes or follicles that survived in each group were utilized for experiments and analysis. Statistical analysis was conducted using the GraphPad Prism 9.0 software (GraphPad Software Inc.). Data are presented as the mean ± standard error of the mean (SEM) for either biological or technical replicates. To compare significance between two groups, a normality test was conducted followed by the unpaired two-tailed Student’s *t* test. The ANOVA test was used for multiple group comparison. *P* < 0.05 was considered statistically significant.

## Results

### MeCP2 protein is highly expressed in primordial and primary follicles, and it is accumulated in aged ovaries

**Fig. 1:**
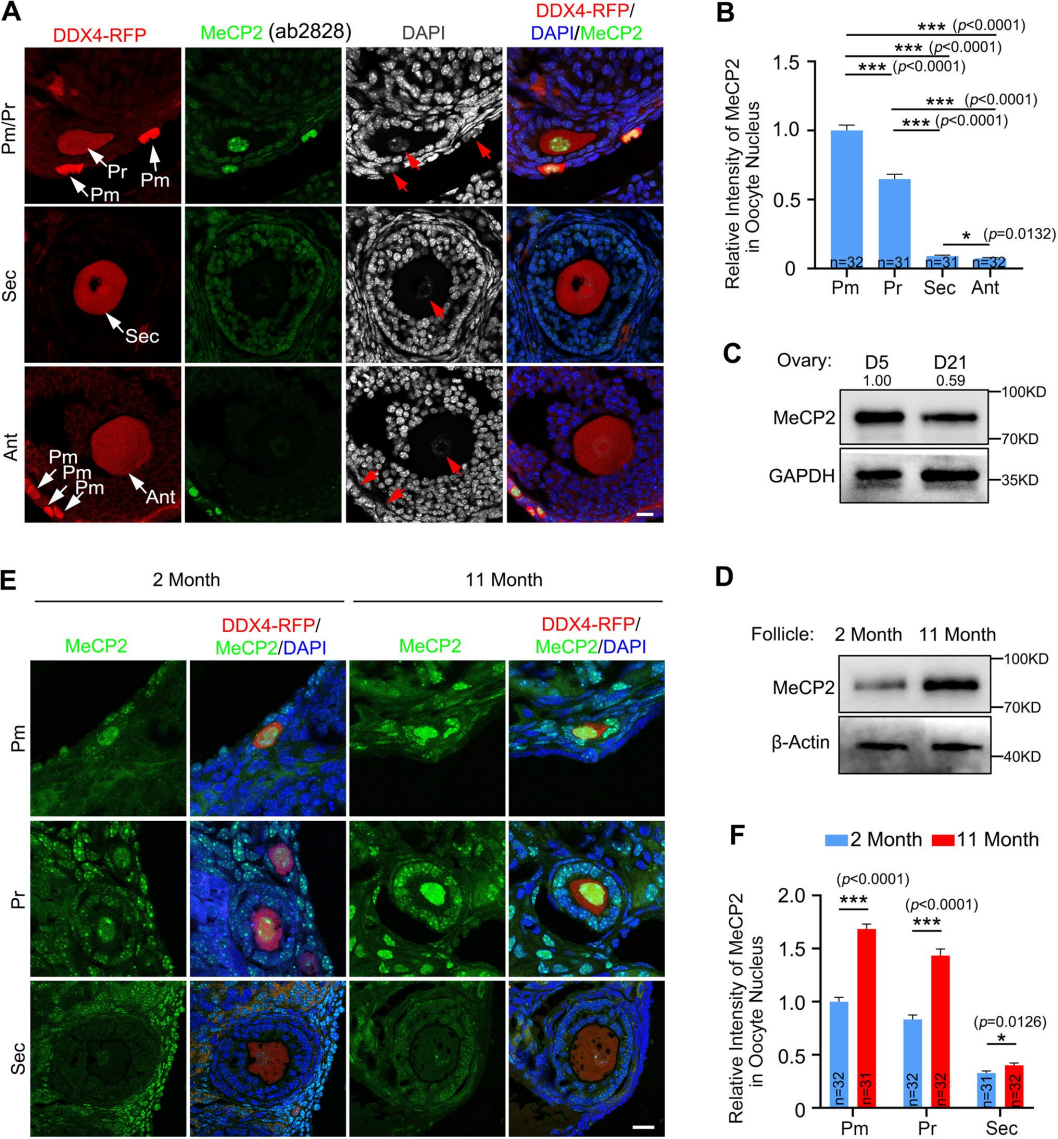
MeCP2 protein is highly expressed in the oocytes of primordial and primary follicles, and expression increases with age. **A** Representative images of follicles at different stages from *Ddx4-Rfp* mice stained with anti-MeCP2 antibody (ab2828). Nuclei are labeled with DAPI (grey or blue). The white arrows indicate oocytes at different stages of the follicle, the red arrows indicate the nucleus of the oocyte. *Pm* primordial follicles, *Pr* primary follicles, *Sec* secondary follicles, *Ant* antral follicles. Scale bar = 20 μm. **B** Quantification of MeCP2 fluorescence intensity in oocytes from indicated stage follicles in (**A**). Data are expressed as means ± SEM (n = 31–32) and analyzed by unpaired two-tailed Student’s *t* test. *P < 0.05; ***P < 0.001. **C** Western blotting displaying the MeCP2 expression in ovaries at postnatal day (PD) 5 and PD 21. GAPDH was blotted as the loading control. **D** Western blotting of follicles derived from wild-type mice at 2 months and 11 months using an anti-MeCP2 antibody. β-Actin was blotted as a loading control. **E** Immunofluorescence staining of ovaries derived from 2-month-old and 11-month-old *Ddx4-Rfp* mice using an anti-MeCP2 antibody. Scale bar = 20 μm. **F** Quantification of the MeCP2 fluorescence intensity in the oocytes from (**E**). Data are shown as means ± SEM (n > 30). Unpaired two-tailed Student’s *t* test. *P < 0.05; ***P < 0.001

Previous studies have demonstrated that transcription directed DNA methylation acquirement during oogenesis [10, 13]. Thus, we assumed that methyl-CpG binding protein 2 (MeCP2), a DNA methylation binding protein, may play a role in the process of oogenesis. To easily observe oocytes in different stage follicles, *Ddx4-Rfp* transgenic mice were utilized wherein a red fluorescent protein (RFP) cassette was inserted after the *Ddx4* promoter [38]. Ovarian sections from *Ddx4-Rfp* mice were utilized for immunofluorescence staining of MeCP2 to characterize the expression pattern of MeCP2 in the ovaries. The MeCP2 protein was observed to be abundant in ovarian primordial and primary follicles, including oocytes and granulosa cells (GCs). However, MeCP2 protein exhibited a significant decrease in secondary follicles, and it remained at a very low level in oocytes of antral follicles (Fig. 1A, B). This was verified using immunostaining with two distinct MeCP2 antibodies (Fig. 1A, B and Supplementary Fig. 1A). Western blotting analysis was conducted to determine the MeCP2 protein levels in mouse ovaries at postnatal day 5 (D5) and day 21 (D21). At day 5, the ovaries contain only primordial follicles and primary follicles, while the first wave of secondary follicles and early antral follicles develop in the ovaries at D21. A decrease of MeCP2 protein levels was observed in the ovaries from D5 to D21 (Fig. 1C). Consistent dynamics of the MeCP2 protein were observed in human ovaries (Supplementary Fig. 1B). However, the mRNA expression of *MeCP2* did not change in oocytes from follicles at different stages (Supplementary Fig. 1C).

In the aged ovary, primordial follicle pools decrease and follicle growth rate declines [48]. *Ddx4-Rfp* mouse ovaries at 2 and 11 months of age were utilized to evaluate the changes of MeCP2 protein levels between young and aged ovaries. The level of the MeCP2 protein exhibited a significant increase in secondary follicles obtained from 11-month-old ovaries (Fig. 1D). The immunofluorescence analysis also indicated a higher expression of MeCP2 protein in primordial and primary follicles from 11-month ovaries compared to those from 2-month ovaries (Fig. 1E). Once the follicle developed into secondary follicle stage, the level of the MeCP2 protein in young ovaries was slightly higher than the aged ones (Fig. 1E, F). Since secondary follicle signifies the completion of follicle growth initiation, we inferred that MeCP2 may suppress follicle growth and that its degradation is essential for follicle growth.

### MeCP2 overexpression impairs follicle growth and survival

**Fig. 2:**
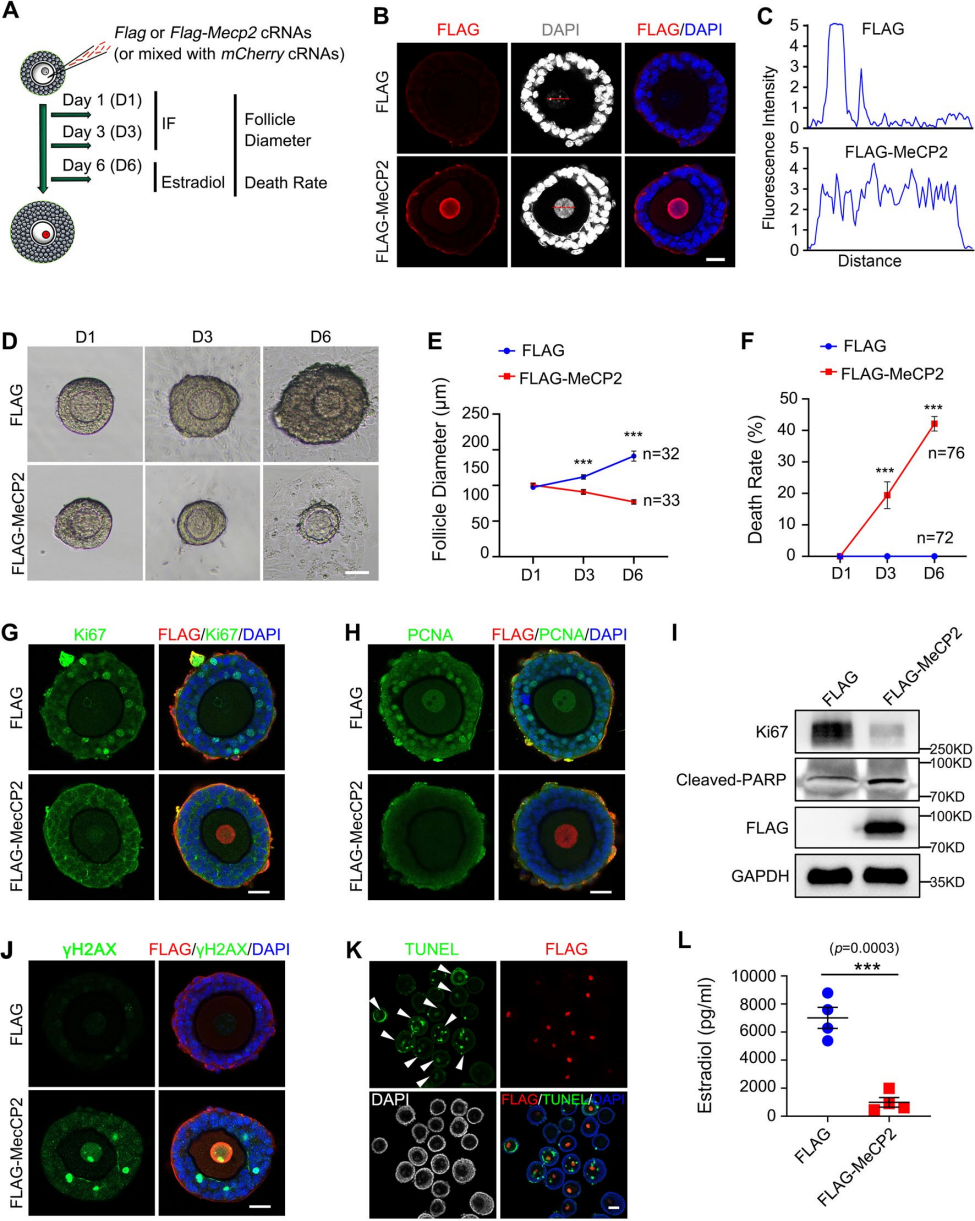
Overexpression of MeCP2 in growing oocytes inhibits follicle growth. **A** A diagram depicting the experimental design. *Flag* or *Flag-MeCP2* cRNAs were microinjected into the oocytes of secondary follicles combined with or without *mCherry* cRNAs and then cultured for 1–6 days. Follicular diameter measurement and mortality statistics were performed on days 1, 3, and 6. Meanwhile, immunofluorescence samples were collected on days 1 and 3. Follicular culture medium was collected on day 6 for hormone determination. **B** Immunostaining showing the effects of MeCP2 overexpression on DNA structure (DAPI) in growing oocytes of secondary follicles. Scale bar, 20 µm. **C** Quantification of DAPI staining signal indicated by the red dotted line. **D** Representative bright-field micrographs of follicle development at days 1, 3, and 6 after *Flag* or *Flag-Mecp2* cRNAs overexpression. Scale bar = 50 μm. **E** Quantification of follicle diameter on days 1, 3, and 6 in the control (n = 32) or MeCP2 overexpression group (n = 33). Unpaired *t* test was used (***P < 0.001). **F** Quantification of follicle death rate on days 1, 3, and 6 in the control or MeCP2 overexpression group (n = 72–76). Unpaired *t* test was used to obtain the *P* value, ***P < 0.001. **G**, **H** Immunofluorescence results for Ki67(**G**) and PCNA (**H**) in the control- and MeCP2-overexpressed oocytes. Scale bar = 20 μm. **I** Western blotting results showing Ki67, Cleaved-PARP, and FLAG expression in the control and MeCP2 overexpression oocytes. GAPDH was used as the protein-loading control. **J** Immunofluorescence results for γH2AX in the MeCP2 overexpression follicular oocytes. Scale bar = 20 μm. **K** Immunofluorescence results for TUNEL in growing oocytes after *Flag* or *Flag-Mecp2* (white arrow heads) cRNAs overexpression within the same image. Scale bar = 20 μm. **L** Estradiol measurements of the follicular culture medium on day 6. The culture medium was pooled in each group. Data are expressed as means ± SEM with four independent experiments (***P < 0.001)

Next, we explored the effects of persistent existence of MeCP2 on follicle development. *FLAG* or *FLAG-MeCP2* cRNAs were microinjected into growing oocytes of secondary follicles, following by culturing the follicles for 3 or 6 days for subsequent analysis (Fig. 2A). The expression of MeCP2 in the primordial follicle was found to be ten-fold higher than in the secondary follicle (Fig. 1A and Supplementary Fig. 1D). As a result, we opted for a concentration of 250 ng/ul cRNA for microinjection, which led to approximately six-fold increase in MeCP2 in the Flag-MeCP2 group compared to the control group (Supplementary Fig. 1E–G). To compare the survival of follicles between two groups, the *mCherry* cRNAs were co-microinjected in the follicle growth study to illustrate the successfulness of the microinjection process. The overexpression of FLAG-MeCP2 was observed in mCherry-positive oocytes, as shown in Supplementary Fig. 1H. The nucleus in the control group displayed several chromatin compartments with clear boundaries and heterogeneous DAPI signal strength, while the chromatin structure in the MeCP2 overexpression group appeared more condensed and uniform (Fig. 2 B-C). Furthermore, MeCP2 overexpression boosted the DAPI signal intensity (Fig. 2B, C). On days 3 and 6, follicles in the control group increased in size (*FLAG* and *mCherry* cRNAs co-injection). In contrast, the overexpression of MeCP2 significantly inhibited follicle growth, resulting in a higher death rate compared with the FLAG group (40% vs 5%, *p* < 0.0001) (Fig. 2D–F). Immunostaining analyses revealed decreased Ki67 and PCNA, two proliferation markers, in oocytes and GCs from early-stage follicles in the MeCP2 overexpression group, which aligns with the delayed follicle growth (Fig. 2G–I). MeCP2 overexpression significantly elevated the signal intensity of γH2AX, cleaved-PARP, and TUNEL in GCs and oocytes (Fig. 2J, K).

Follicle growth is dependent on communication between oocyte and GCs, such as oocyte-secreted factors, gap-junctions, oocyte-derived microvilli and GCs-derived F-actin-containing transzonal projections (TZPs) [4, 49, 50]. The intensity of TZPs was significantly decreased in follicles overexpressing MeCP2 (Supplementary Fig. 1I). Subsequent to the overexpression of MeCP2, a notable decrease in the mRNA levels of *Fshr*, *Amhr*, and *Inhibin* genes, which are known for their high expression in granulosa cells, was observed (Supplementary Fig. 1 J). We also measured the estradiol (E2) concentration in follicle culture medium, which is synthesized and secreted by granulosa cells. The E2 level in the FLAG-MeCP2 group is 2000 pg/ml, which is significantly lower than the average E2 level of 7000 pg/ml in the FLAG group (Fig. 2L). Collectively, these results indicated MeCP2’s accumulation in oocytes inhibits follicle growth and induced DNA damage and apoptosis in both oocytes and GCs. That is, MeCP2 degradation is essential for oocyte survival and follicle growth.

### MeCP2 overexpression induced transcription dysregulation in growing oocytes

**Fig. 3:**
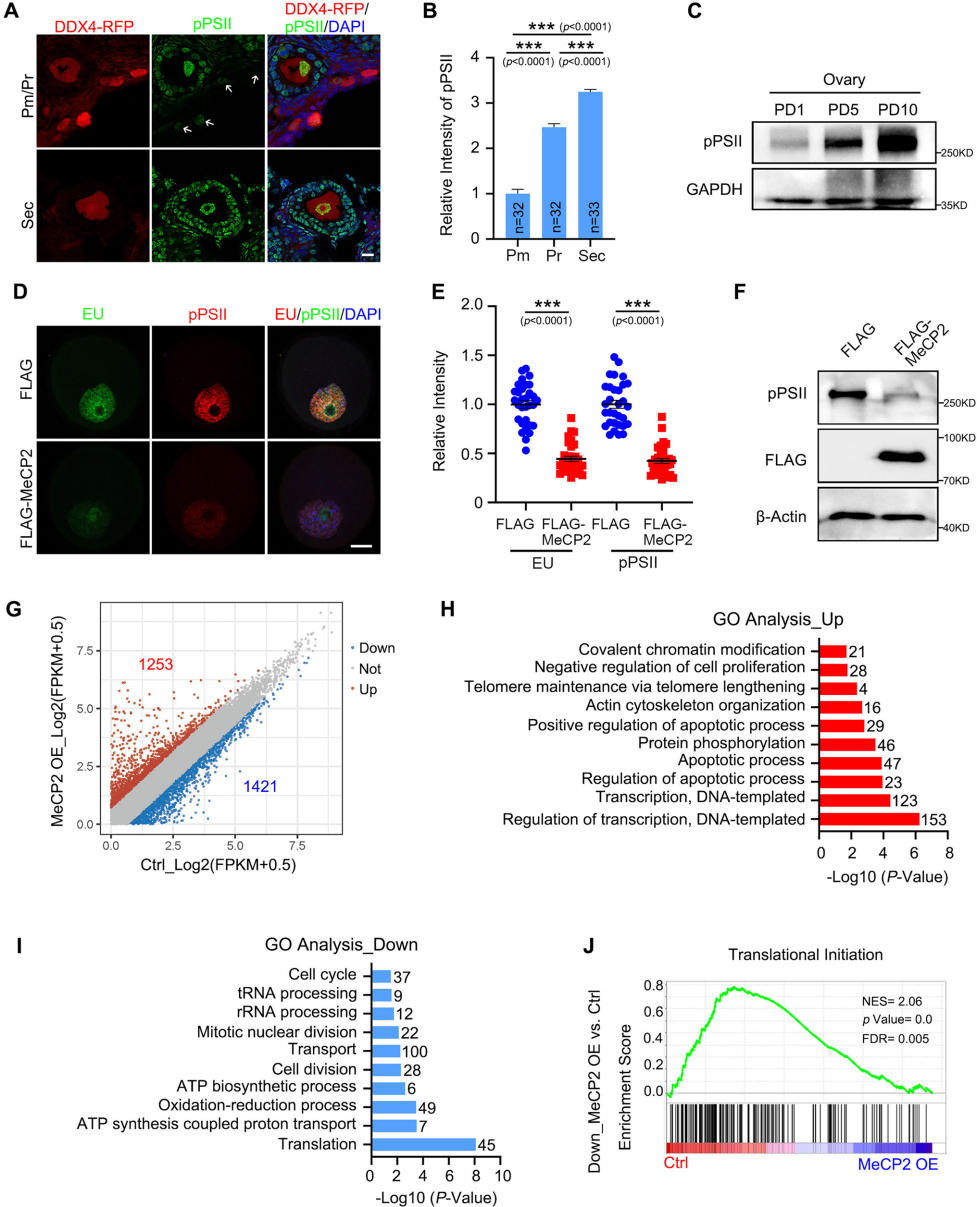
MeCP2 overexpression induced transcription dysregulation in growing oocytes. **A** Immunofluorescence staining of pPSII in different stages of follicular oocytes from *Ddx4-Rfp* mice. The white arrows indicate the primordial follicles. Scale bar = 20 μm. pPSII, phosphorylated RNA polymerase II at Ser 5 in the C-terminal domain. **B** Quantification of pPSII fluorescence intensity in oocytes from (**A**). Data are shown as means ± SEM (n = 32–33). Unpaired two-tailed Student’s *t* test. ***P < 0.001. **C** Western blotting of pPSII in ovaries at postnatal day (PD) 1, 5, and 10. GAPDH was blotted as the loading control. **D** Immunostaining images of oocyte after *Flag* or *Flag-Mecp2* mRNAs overexpression for 1 day with anti-pPSII and 5-Ethynyl uridine (EU) to indicate the RNA transcription activity in oocytes. Scale bar = 10 μm. **E** Quantification of pPSII and EU fluorescence signal intensity in (**D**). Data are presented as means ± SEM (n = 31–32). Unpaired two-tailed Student’s *t* test. ***P < 0.001. **F** Western blotting results showing the pPSII protein level in growing oocytes after overexpression of *Flag* or *Flag-Mecp2* mRNAs. β-Actin was blotted as the loading control. **G** Scatter plot exhibiting the transcriptional changes between control (Ctrl) and MeCP2 overexpression (MeCP2 OE) oocytes. Gene expressions increased or decreased by more than two-fold are indicated in red and blue, respectively. FPKM, fragments per kilobase per million mapped reads. **H**, **I** Gene Ontology (GO) analysis of the upregulated (**H**) and downregulated (**I**) in the MeCP2 overexpression oocytes for the enriched biological process. The transcripts numbers of each biological process are labeled on the right panel. **J** GSEA enrichment plots of the indicated signaling pathways and boxplots of key component genes in the MeCP2 overexpression oocytes

Since MeCP2 has been characterized as both a transcription activator and repressor [22], we examined the effect of MeCP2 overexpression on transcription in growing oocytes. The level of phosphorylated RNA polymerase II at Ser 5 in the C-terminal domain (referred to as pPSII), an indicator of transcription activity, was gradually increased from primordial to secondary follicles (Fig. 3A, B). The pPSII level were assessed in the ovaries on day 1, day 5, and day 10, corresponding to the first waves of primordial follicles, primary follicles, and secondary follicles developed, respectively. An elevated level of pPSII protein level was observed during follicle growth (Fig. 3C), whereas MeCP2 exhibited a trend of degradation (Fig. 1A–C). Then, we cultured follicles after microinjecting *FLAG* or *FLAG-MeCP2* cRNAs into oocytes, and the oocytes were isolated one day later. The uridine analog 5-ethynyluridine (EU) was then incorporated into newly transcribed RNA to monitor transcription activity prior to fixation. We observed that the overexpression of MeCP2 led to a significant decrease in pPSII and EU signal (Fig. 3D, E, Supplementary Fig. 1 K). The western blot analysis confirmed that the MeCP2 overexpression group exhibited a lower pPSII level than the control group (Fig. 3F).

To characterize the genes regulated by MeCP2, we performed RNA-seq on growing oocytes following *FLAG* or *FLAG-MeCP2* microinjection in oocytes of secondary follicles. We identified approximately 10,000 transcripts with the FPKM > 1 (Supplementary Table 4). A total of 1253 transcripts were found to be up-regulated, while 1421 genes were down-regulated, based on a twofold change threshold (Fig. 3G). The Gene Ontology (GO) analysis revealed that the up-regulated mRNA transcripts encoded proteins related to the regulation of transcription, transcription, apoptosis, and covalent chromatin modification (Fig. 3H). The genes that are down-regulated after MeCP2 overexpression are primarily associated with translation and ATP synthesis (Fig. 3I). The Gene Set Enrichment Analysis (GSEA) confirmed that the overexpression of MeCP2 inhibited gene expression related to translational initiation (Fig. 3J). These results indicated that the overexpression of MeCP2 induced transcription dysregulation in growing oocytes.

### Overexpression of MeCP2 induced DNA hypermethylation in growing oocytes

**Fig. 4:**
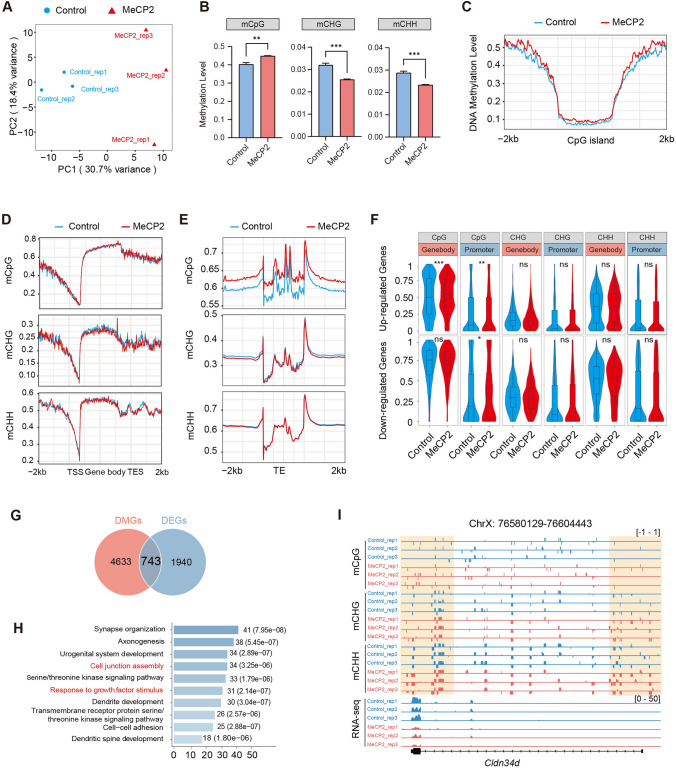
Overexpression of MeCP2 in growing oocytes leads to DNA hypermethylation in CpG islands. **A** PCA showing consistency between the three biological replicates of whole-genome bisulfite-sequencing (WGBS) oocyte samples of Control and Mecp2 overexpression. **B** Methylation levels of mCpG, mCHG, and mCHH across oocyte samples in control and MeCP2 overexpression groups. Unpaired two-tailed Student’s *t*-test. **P < 0.01. ***P < 0.001. **C** Density plot showing the average DNA methylation is increased around bivalent CpG islands in MeCP2-overexpressed growing oocytes. **D** Methylation level of mCpG, mCHG, and mCHH within gene body and 2 kb flanking (upstream and downstream) regions in growing oocytes from control and MeCP2-overexpressed groups. **E** Methylation level of mCpG, mCHG, and mCHH within transposable elements (TE) and 2 kb flanking regions in growing oocytes from control and MeCP2-overexpressed groups. **F** Methylation level of mCpG, mCHG, and mCHH in gene body and promoter region of up-regulated and down-regulated genes after MeCP2 overexpression. **G** The Venn diagram showing the intersection of differential expression gene (DEGs) and DMR-associated genes (DMGs). **H** The biological process pf Gene ontology (GO) annotation of intersection genes in (**G**). **I** Integrated Genome Browser view of *Cldn34d* gene with increased methylation level and down-regulated mRNA after MeCP2 overexpression

DNA methylation is scarce in the oocyte of the primordial follicle and is gradually established as oocyte growth [13]. Since MeCP2 exhibited the highest expression in primordial follicles and significantly decreased in growing oocytes (Fig. 1A), indicating excess of MeCP2 may have a detrimental effect on the establishment of DNA methylation during oogenesis. To evaluate the hypothesis, we determined the effect of MeCP2 overexpression on DNA methylation in growing oocyte. *FLAG* or *FLAG-MeCP2* cRNAs were microinjected into growing oocytes obtained from the ovaries of D14 mice. After a 24-h culture period, the oocytes were subjected to low-input whole-genome bisulfite sequencing (WGBS). Each group was sequenced using three biological replicates (referred as Control and MeCP2). Approximately 80% of the reads were successfully mapped to the reference genome in each sample. When conducting a comparison of genome-wide profiles of DNA methylation in growing oocytes through principal component analysis, it was observed that MeCP2 overexpression formed a distinct cluster separate from the control oocytes (Fig. 4A). As reported previously, the methylated cytosines can be divided into CpG (CG) and non-CpG (CHG and CHH), and both CpG and non-CpG sits are gradually methylated by DNMTs during oocyte growth [11]. The level of CG methylation is higher in oocytes with MeCP2 overexpression compared to the control, while the levels of CHG and CHH methylation were decreased (Fig. 4B and Supplementary Fig. 2A). In oocytes overexpressing MeCP2, the level of methylated CpG (mCG) in CpG islands and their flanking regions is higher compared to control oocytes (Fig. 4C). A total of 45,234 differentially methylated regions (DMRs) are identified, with over 35,000 DMRs (233,863 in exons and 12,032 in promoters) exhibiting hypermethylation and only 8000 DMRs (6,156 in exons and 2,148 in promoters) displayed hypomethylation (Supplementary Fig. 2B). Then, we calculated the DNA methylation levels of CG, CHG, and CHH for both genes and transposable elements (TEs). The methylation level of the CpG islands was slightly increased in the gene body and 5′ flanking regions (Fig. 4D), as well as mildly increased in TE body and flanking regions (Fig. 4E). In contrast, the methylation level of CHG is decreased in gene and TE regions, while the level of CHH methylation remains comparable (Fig. 4D, E). We also found that the methylation levels of all the CG sub-contexts (CGA, CGG, CGC, and CGT motifs) in MeCP2-overexpressing oocytes were higher than those in control oocytes (Supplementary Fig. 2C). Among, The CAG motif within the CHG sub-contexts exhibited a lower methylation level compared to the CCG and CTG motifs (Supplementary Fig. 2D). However, the majority of sub-contexts within CHH demonstrated comparable methylation levels between the two groups (Supplementary Fig. 2E).

Next, we integrated the RNA-seq and WGBS data for analysis. We analyzed the methylation levels of 1253 up-regulated genes and 1421 down-regulated genes after overexpression of MeCP2. In the upregulated genes, only the CpG methylation level exhibited an increase in both the gene body and promoter (Fig. 4F), as shown in the Genome Browser in Supplementary Fig. 2F. In the genes that were down-regulated, the CpG methylation within the gene body was comparable in two groups. However, there was an increase in the CpG methylation in the promoter (Fig. 4F). The levels of CHG and CHH methylation in both up-regulated and down-regulated genes were comparable (Fig. 4F). The 45,234 DMRs are annotated with 5376 differential methylated genes (DMGs), with approximately 743 genes overlapping between the DMGs and differential expressed genes (DEGs) (Fig. 4G). The GO analysis revealed that the 743 genes affected by methylation were enriched in process related to synapse organization, cell junction assembly, and response to growth factor stimulus (Fig. 4H). For instance, *Cldn34d*, a gene predicted to be involved in bicellular tight junction assembly and cell adhesion, was down-regulated in oocytes overexpressing MeCP2 with elevated DNA methylation levels (Fig. 4I).

### CRL4^DCAF13^ targets MeCP2 for ubiquitination and degradation in HeLa cells

**Fig. 5:**
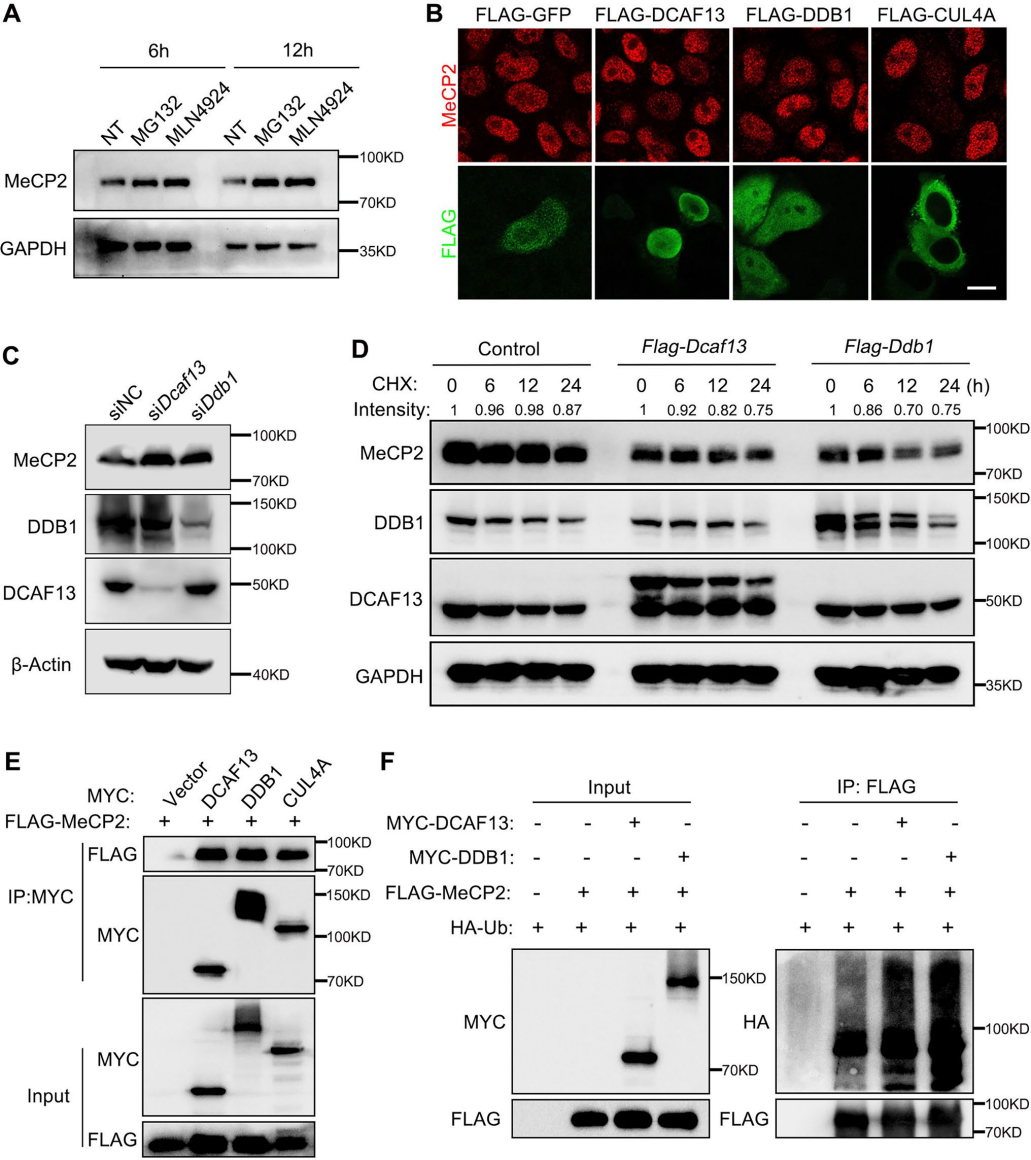
MeCP2 is poly-ubiquitinated and degraded by CRL4^DCAF13^ E3 ligase. **A** Western blotting results showing the MeCP2 protein level in Hela cells after 10-μM MG132 or MLN4924 treatment for 6 h and 12 h. GAPDH was blotted as the loading control. **B** Immunofluorescence results for inner MeCP2 and FLAG in Hela cells after transfection with FLAG-GFP, FLAG-DCAF13, FLAG-DDB1, and FLAG-CUL4A. Scale bar = 10 μm. **C** Western blotting results for the indicated proteins from Hela cells after transfection with siRNAs-targeting negative control (siNC), *Dcaf13* (si*Dcaf13*), and *Ddb1*(si*Ddb1*). β-Actin was blotted as the loading control. **D** Western blotting results demonstrating the MeCP2, DDB1, and DCAF13 expression in Hela cells transfected with control, FLAG-DCAF13, and FLAG-DDB1 plasmids followed by CHX disposing at 0, 6, 12, and 24 h, respectively. GAPDH was blotted as the loading control. **E** Immunoprecipitation and Western blotting results showing the interaction among MeCP2 and DCAF13, DDB1, and CUL4A in Hela cells. **F** IP followed by Western blotting showing MeCP2 polyubiquitination in the control HeLa cells and in cells transfected with plasmids encoding the indicated proteins

Based on our results, we speculated that the degradation of MeCP2 may be a necessary condition for follicle growth. We investigated the regulatory mechanism of MeCP2 protein degradation. Given the pivotal role of the Cullin4-RING (CRL4) E3 ligase in the maintenance of ovarian follicles [34, 51], we hypothesized that the CRL4 E3 ligase may mediate MeCP2’s degradation. As expected, treatment of HeLa cells with proteasome inhibitor MG132 or CRL4 E3 ligase inhibitor MLN4924 increased MeCP2 protein level at 6 h and 12 h (Fig. 5A). The mRNA levels of several DDB1-Cul4 associated factors (DCAFs), which are responsible for recognizing substrate for degradation, were examined, in the reported RNA-seq data of human and mouse oocytes (GSE107746 and GSE135787) [52, 53]. Several DCAFs were identified to be expressed in growing oocytes, and DCAF13 exhibited the highest expression in secondary follicle oocytes in both human and mouse (Supplementary Fig. 3A, B). The FLAG-tagged DCAFs were transiently transfected into HeLa cells to determine the mRNA and protein levels of MeCP2. We found that the overexpression of DCAF13 significantly decreased MeCP2 protein level, while not affecting the mRNA level (Supplementary Fig. 3C, D). Ectopic overexpression of DDB1, CUL4A, and DCAF13 decreased inner MeCP2 protein level, as demonstrated by immunofluorescence and immunoblotting (Fig. 5B and Supplementary Fig. 3E). Overexpression of DCAF13-induced MeCP2 decrease was blocked by treatment with the proteasome inhibitor (MG132). This suggested that the degradation of MeCP2 protein was dependent on DCAF13 and proteosome-dependent degradation pathway. RNA interference of *Ddb1* and *Dcaf13* caused an increase in the MeCP2 protein level (Fig. 5C). When HeLa cells overexpressing DDB1 and DCAF13 were treated with CHX, an inhibitor of protein synthesis, the degradation rate of MeCP2 increased compared to the control (Fig. 5D). We confirmed that MeCP2 directly interacts with the CRL4 E3 ligase through co-immunoprecipitating of key components of the CRL4 E3 ligase complex (CUL4A and DDB1) and the substrate adaptor DCAF13 (Fig. 5E). Then, we performed an in vivo ubiquitination assay and found overexpression of DDB1 and DCAF13 led to an increase in MeCP2 ubiquitination (Fig. 5F). Collectively, these results indicated that the CRL4^DCAF13^ E3 ligase is responsible for targeting MeCP2 for ubiquitination and subsequent degradation.

### Dcaf13 deletion in oocytes caused MeCP2 protein accumulation and inhibited follicle growth

**Fig. 6:**
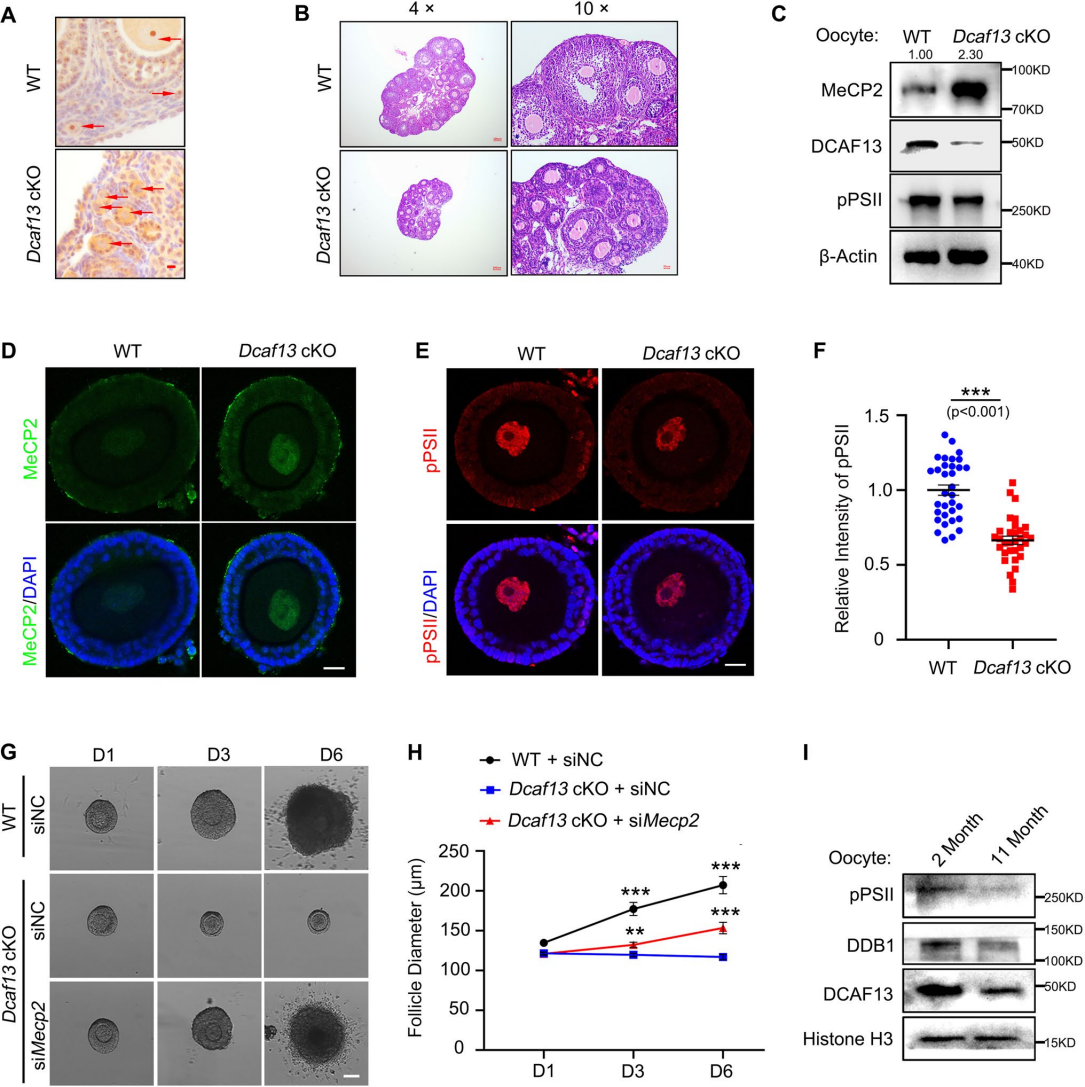
Deletion of *Dcaf13* caused MeCP2 accumulation and transcription dysregulation in growing oocytes. **A** The representative images showing the immunohistochemistry of DCAF13 in ovaries from WT (*Dcaf13*^*f/f*^) and *Dcaf13* cKO (*Dcaf13*^*f/f*^*; Gdf9-Cre*) mice at postnatal day 21. Scale bar = 20 μm. The red arrows indicate oocytes of follicles. **B** The representative H&E staining images of ovaries from WT (*Dcaf13*^*f/f*^) and *Dcaf13* cKO (*Dcaf13*^*f/f*^*; Gdf9-Cre*) mice at postnatal day 21. The 4 × and 10 × objectives were used. Scale bars are labeled in each image. **C** Western blotting results showing the MeCP2, pPSII, and DCAF13 protein levels in oocytes from WT and *Dcaf13* cKO mice. β-Actin was blotted as the protein-loading control. **D** Immunofluorescence for MeCP2 expression in secondary follicles derived from WT and *Dcaf13* cKO mice. Scale bar = 20 μm. **E** Immunofluorescence results for the pPSII expression in the nucleus of follicular oocyte derived from WT and *Dcaf13* cKO mice. Scale bar = 20 μm. **F** Quantification of the pPSII fluorescent signal intensity in (**E**). Data are expressed as means ± SEM (n = 32–33). Significance was determined using unpaired two-tailed Student’s *t* test. ***P < 0.001. **G** Representative bright-field images showing the follicle development at days 1, 3, and 6 after siNC or si*Mecp2* microinjection in secondary follicles from WT and *Dcaf13* cKO mice (n = 2 mice). Scale bar = 100 μm. **H** Quantification of follicle diameter in (**G**) in the indicated group. Data are presented as means ± SEM (n = 31–38). Two-tailed Student’s *t* test. **P < 0.01, ***P < 0.001. **I.** Western blotting results showing the pPSII, DDB1, and DCAF13 expression in oocytes derived from 2- and 11-month-old mice. Histone H3 was blotted as the loading control

We previously generated *Dcaf13* conditional knockout (*Dcaf13* cKO) mice specifically in oocyte from primordial follicle stage by crossing *Dcaf13*^*f/f*^ and *Gdf9-Cre* mice [6]. The *Dcaf13* cKO mice (*Dcaf13*^*f/f*^*; Gdf9-Cre*) were infertile exhibition with delayed oocyte growth and meiosis disorder [6, 37]. DCAF13 proteins were expressed low in the primordial follicle and gradually increased in the growing follicle, with the opposite expression profile of MeCP2 (Fig. 6A). The level of DCAF13 proteins in oocytes were significantly diminished in *Dcaf13* cKO mice, as illustrated in Fig. 6A. The first waves of ovarian follicles develop into early antral follicles in 3-week-old mice, while *Dcaf13* deletion in oocytes caused the majority of follicles growth arrest at the secondary follicle stage (Fig. 6B). Only a small proportion of follicles prior to 4 weeks were able to develop into antral follicles with the stimulation of PMSG in *Dcaf13* cKO mice. *Dcaf13* deletion, similar to MeCP2 overexpression, resulted in a high level of DNA damage and apoptosis, as indicated by the staining of γH2AX, cleaved caspase 3, and cleaved PARP (Supplementary Fig. 4A–C). Consistent with the results in HeLa cells shown in Fig. 5C, *Dcaf13* deletion significantly caused MeCP2 increase in growing oocytes (Fig. 6C, D). Meanwhile, it also resulted in attenuated transcription in growing oocyte of the secondary follicle (Fig. 6C, E and F).

*MeCP2* knockdown was conducted in *Dcaf13* cKO follicles to confirm the impact of DCAF13 loss on follicle growth through the accumulation of MeCP2. In contrast to the sustained growth of follicles in wild-type (*Dcaf13*^*f/f*^) mice, *Dcaf13* cKO follicles exhibited only minimal growth (Fig. 6G, H). In contrast, the partial rescue of the *Dcaf13* deletion-induced follicle growth defects was observed upon *MeCP2* knockdown (Fig. 6G, H). The knockdown efficiency of *MeCP2* was validated by RT-PCR (Supplementary Fig. 4D). Together, these results demonstrate that MeCP2 is a substrate of CRL4^DCAF13^ E3 ligase, and the function of CRL4^DCAF13^ in sustaining oogenesis and follicle growth is, to some extent, accomplished through degradation of MeCP2.

We detected the protein levels of pPSII and CRL4 components in growing oocytes derived from 2- month-old and 11-month-old mice. The levels of DDB1, DCAF13, and pPSII were significantly decreased in the aged ovary (Fig. 6I). In contrast, the protein level of MeCP2 exhibited significant increase in aged ovary (Fig. 6I). The deficiency of CRL4^DCAF13^ might result in a MeCP2 increase in aged oocytes (Fig. 1D–F), offering a potential explanation for follicle arrest in the aging ovary. These results indicate that the aberrant DCAF13-MeCP2 axis causes transcription dysregulation and halts follicle growth.

### Abnormal DCAF13-MeCP2 axis repressed translation and activated genes expression relative to chromatin covalent modification

**Fig. 7:**
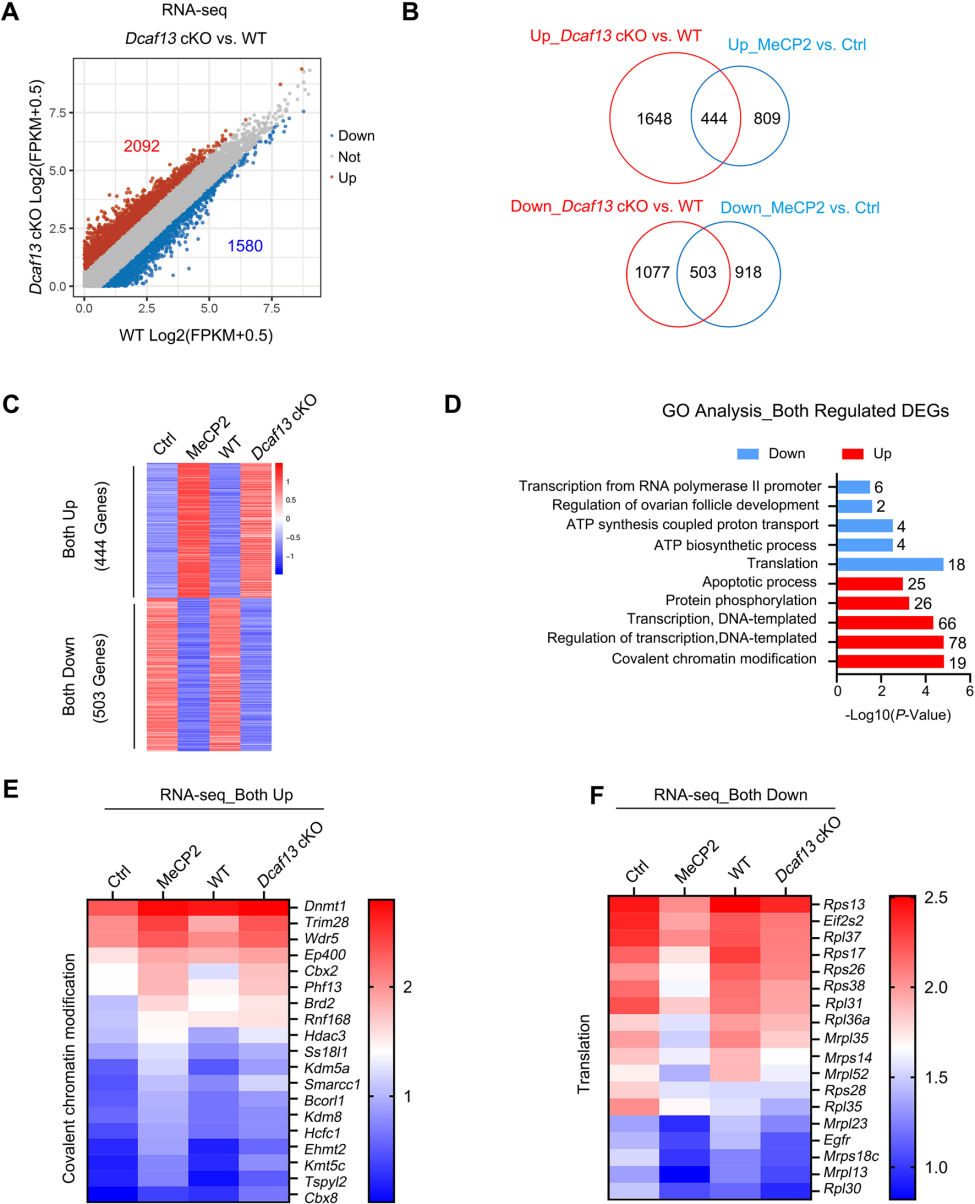
Aberrant DCAF13-MeCP2 axis induced genes expression abnormality in growing oocytes. **A** Scatter plot displaying the transcriptional changes between wild-type (WT, *Dcaf13*^*f/f*^) and *Dcaf13* cKO (*Dcaf13*^*f/f*^; *Gdf9-Cre*) oocytes. Gene expressions that were increased or decreased by more than two-folds are indicated in red and blue, respectively. FPKM, fragments per kilobase per million mapped reads. **B** The upper Venn diagram showing the *Dcaf13* cKO-upregulated genes overlapped with the MeCP2 overexpression-upregulated genes, while the *Dcaf13* cKO downregulated genes overlapped between the MeCP2 overexpression downregulated genes (lower panel). **C** The heatmap showing the both down- and up-regulated genes in growing oocytes after *Dcaf13* cKO or MeCP2 overexpression relative to each control. **D** The enriched biological process of both downregulated and upregulated in MeCP2 overexpression oocytes overlapped between *Dcaf13* cKO oocytes obtained via Gene Ontology (GO) analysis. The transcripts numbers of each biological process are labeled on the right panel. **E**, **F** Heatmaps displaying the expression dynamics of both upregulated (**E**) and downregulated (**F**) genes from the MeCP2 overexpression oocytes overlapping between *Dcaf13* cKO oocytes

**Fig. 8:**
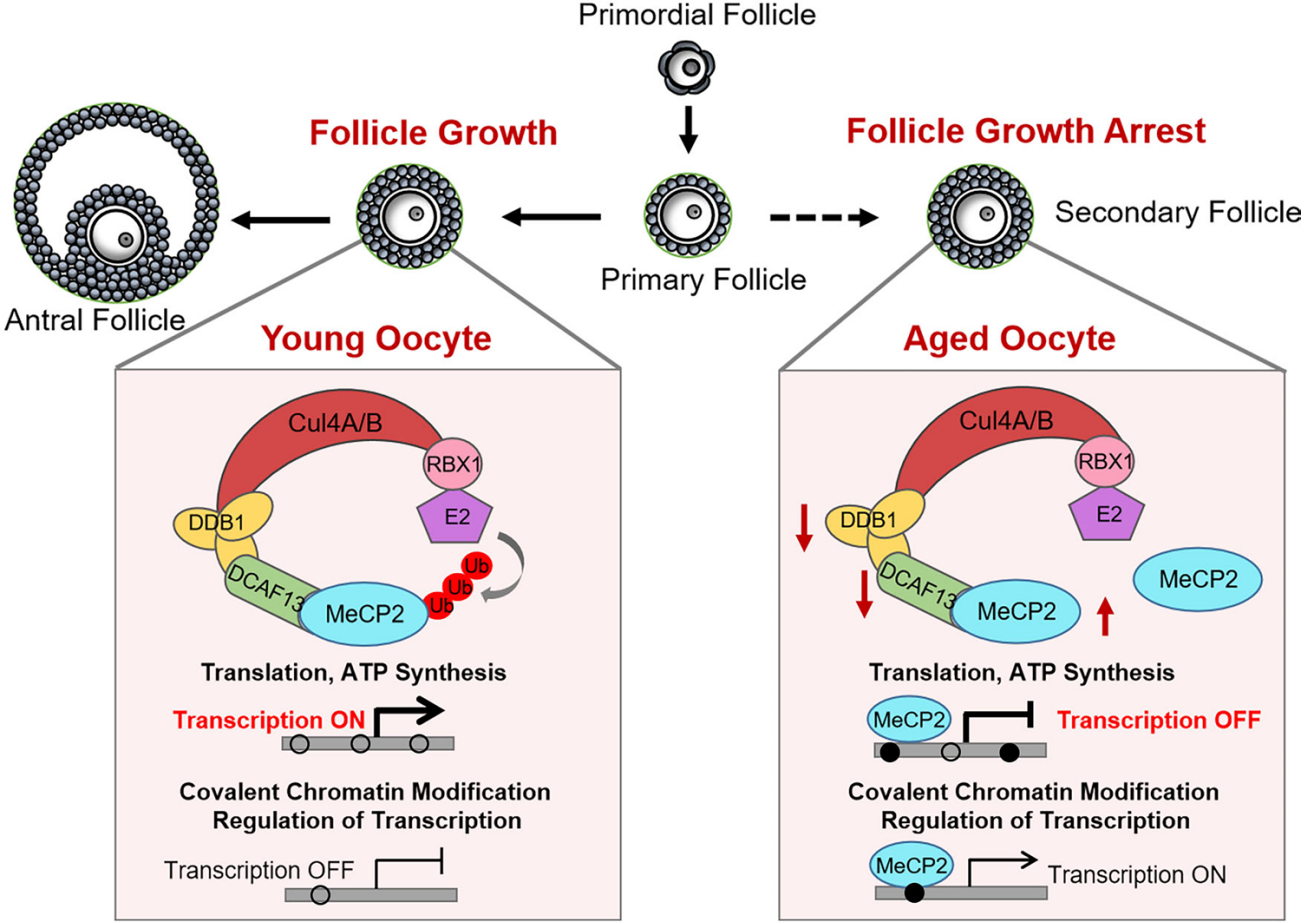
The schematic illustration of the DCAF13-MeCP2 axis involved in follicle growth. In the secondary follicles, MeCP2 is polyubiquitinated and degraded by CRL4^DCAF13^ E3 ligase in young oocytes. This process promotes gene expression associated with translation and ATP synthesis, while inhibiting gene expression related to chromatin modification. As a result, successful development from the secondary follicle to antral follicle is ensured. In aged oocytes, decreased levels of DDB1 and DCAF13 stabilize MeCP2, resulting in DNA hypermethylation in CpG islands, transcription dysregulation, and follicle growth arrest

We also used RNA-seq to identify important and conserved transcripts in the DCAF13-MeCP2 axis using growing oocytes from wild-type and *Dcaf13* cKO mice. We identified upregulated (n = 2092) and downregulated (n = 1580) genes after *Dcaf13* deletion (Fig. 7A and Supplementary Table 5). Through Venn analysis, approximately 1/3 (444 out of 1253 genes) of the upregulated transcripts following MeCP2 overexpression overlapped with those that were upregulated after *Dcaf13* cKO (Fig. 7B). Approximately 500 genes, 1/3 of which were present in both groups, exhibited overlap in the downregulated genes with *Dcaf13* cKO and MeCP2 overexpression (Fig. 7B, C). The GO analysis of co-regulated genes revealed that the abnormal DCAF13-MeCP2 axis in growing oocyte repressed genes expression related to translation and ATP synthesis, while it activated gene expression associated with chromatin covalent modification, regulation of transcription, and the apoptotic process (Fig. 7C–E). The upregulated genes associated with chromatin covalent modification and down-regulated genes related to translation were validated by RT-PCR after MeCP2 overexpression (Supplementary Fig. 4E, F). These results suggested that the DCAF13-MeCP2 axis is essential for normal gene expression in growing oocytes (Fig. 8).

## Discussion

The DNA binding protein Methyl-CpG-binding protein 2 (MeCP2) recognizes methylated DNA [24, 54]. Extensive research in brain development has provided a great deal of information about the functional role of MeCP2 [23]. Although MeCP2 is widely expressed, its role in other tissues, especially in the ovary, is poorly understood. Our findings show that MeCP2 is highly expressed in quiescent follicles and that the MeCP2 protein is quickly degraded when follicular growth begins. This study firstly demonstrated that the degradation of MeCP2 by CRL4^DCAF13^ E3 ligase is required in growing oocyte to maintain normal DNA methylation transcription and follicle survival.

Ovarian aging is characterized by the gradual depletion of ovarian follicles and reduced ability to produce oocytes competent for fertilization and further development [55]. Abnormal DNA methylation is associated with aging of the ovary and oocyte [56–58]. For instance, deficiency of ten-eleven translocation (TET) proteins, specifically TET1 and TET2, resulted in increased DNA methylation because of the abnormal conversion of 5-methylcytosine (5mC) to 5-hydroxymethylcytosine (5hmC), ultimately leading to declined follicle reserve and premature ovarian failure [59, 60]. We found that MeCP2 levels in aged primordial and primary follicles are much higher than in young mice ovaries. Additionally, overexpression of MeCP2 induced DNA hypermethylation and follicle atresia, consistent with *Tet1* or *Tet2* deletion.

Recent studies have demonstrated that MeCP2 is an organizer of heterochromatin by competing with histone H1 to regulate nucleus liquid–liquid phase separation [61]. The chromatin structure of the oocyte changes dynamically during folliculogenesis and oogenesis. The nuclei of oocyte in the primordial and primary follicles exhibited compact structure, while the chromatin in growing oocytes appears relatively less condensed, as evidenced by the DAPI signal staining (Fig. 1A). MeCP2 overexpression resulted in more uniform and increased DAPI signal in growing follicles (Fig. 2A). The significance of DAPI signal after MeCP2 overexpression may indicate condensed chromatin. However, further experiments are necessary to confirm this conclusion in future studies. Moreover, the role of MeCP2 in the primordial follicle seem to be more important due to its highest protein level. While it was hypothesized that MeCP2 is crucial for preserving the condensed chromatin structure in quiescent follicles, this study did not offer direct evidence, leaving it for future investigation. Recent research found that MeCP2-induced heterochromatin assembly was hampered by DNA methylation [62]. This might provide insights into the relationship between low DNA methylation and high MeCP2 levels in primordial follicles.

We found an exclusive pattern between MeCP2 and DNA methylation in oogenesis when the MeCP2 protein should be degraded during DNA methylation establishment. This implies that the continued presence of MeCP2 in growing oocytes may be deleterious for the establishment of de novo DNA methylation. We found that the overexpression of MeCP2 induced global DNA hypermethylation, potentially causing dysregulated transcription or indicating a reciprocal causation. The widely accepted notion is that transcription drives de novo DNA methylation, a conclusion drawn from high-throughput sequencing data obtained from mouse and human oocytes [13]. Apart from the accumulation of *Dnmt3a, Dnmt3l, and Dnmt1* mRNAs, transcription-coupled histone modification and chromatin accessibility also affect initiation of DNA methylation [63, 64]. However, there has been limited investigation into the mechanism by which the gradual increase in DNA methylation in oocytes marginally affected transcription. Our study firstly elucidated that MeCP2 degradation is responsible for preventing DNA hypermethylation in CpG islands in growing oocytes. This study provided direct evidence that the degradation of MeCP2 is required for normal de novo DNA methylation, transcription, and follicle growth. Further analysis is required to elucidate the relationship between DMRs affected by MeCP2 overexpression and MeCP2-binding sites in non-growing and growing oocytes.

Our study demonstrated that MeCP2 overexpression significantly caused transcription dysregulation (Fig. 3D–F). The RNA-seq data, however, revealed a similar number of up-regulated and down-regulated genes in growing oocytes (Fig. 3G). This result is consistent with previous studies on *MeCP2* mutations in brain development, which similarly demonstrated alterations in the expression of genes, both up-and-down-regulated genes [22]. It was commonly accepted that MeCP2 functions as a transcriptional repressor by binding methylated DNA at CpG islands to repress transcription [19, 20]. A recent study also demonstrated that MeCP2 repressed the rate of transcriptional initiation in long genes with significant methylation [65]. Several recent studies have shown that MeCP2 is capable of activating genes transcription and binding to numerous non-methylated loci, in addition to methylated CpG [18, 22]. This may provide an explanation for the RNA-seq results.

DCAF13, encoded by an evolutionarily conserved gene, is predominantly localized in the nucleus and nucleolus, can participate in rRNA processing since it possesses a SOF1 domain [6]. DCAF13 function as a substrate recognizer of the CRL4 E3 ligase. We previously found that deletion of mouse *Dcaf13* gene results in morula arrest and blastocyst formation failure due to H3K9me3 accumulation and defective SUV39H1 degradation in early embryos [35]. DDB1 and DCAF13 exhibit high expression levels in oocytes (Figure S2A, B). Deletion of *Ddb1* in oocytes caused DNA hypermethylation and follicle growth arrest. It was demonstrated that the CRL4^DCAF1^ E3 ligase activates the catalytic activities of TET1-3 through mono-ubiquitination in oocytes [34]. This study revealed that MeCP2 is a substrate of CRL4 E3 ligase through DCAF13 for proteolysis, and MeCP2 overexpression also induced DNA hypermethylation. Given DDB1's ability to interact with numerous DCAFs for substrate recognition, this study may further supplement evidence for *Ddb1*-deletion-induced DNA hypermethylation through CRL4-DDB1-DCAF13-MeCP2 axis. Notably, during oocyte growth, MeCP2 is not the only one substrate of CRL4^DCAF13^ E3 ligase. PTEN has been previously identified as a substrate of the CRL4^DCAF13^ E3 ligase during oocyte growth [37, 66]. Since successful oogenesis requires many biological events, MeCP2 and PTEN may be cooperatively degraded by CRL4^DCAF13^ E3 ligase. However, MeCP2, DDB1, and DCAF13 co-existed in the primordial follicle. This suggests the presence of an alternative signaling pathway that activates the MeCP2 degradation process of CRL4^DCAF13^ E3 ligase in growing oocytes. This is an interesting theme to be investigated in future. Another aspect requiring clarification in this study is the mechanism by which MeCP2 in oocytes induces follicle atresia. We supposed that MeCP2 might affect gene expression to block the communication between the oocyte and GCs. We found GDF9 and BMP15 were not changed at both the mRNA and protein level after MeCP2 overexpression (data not shown). The genes affected by MeCP2 relative to gap junction assembly (Fig. 4H) might provide valuable insights into the oocyte-GCs crosstalk. Further investigation is needed to understand the mechanism by which MeCP2 represses follicle growth.

This study initially discovered MeCP2 protein is highly expressed in quiescent follicles and rapidly disappeared in growing oocytes. The continued presence of MeCP2 in growing oocyte has deleterious effect on the de novo DNA methylation establishment. MeCP2 protein could be rapidly degraded by the CRL4^DCAF13^ E3 ligase in growing oocytes to facilitate follicle growth. Moreover, the decrease of DDB1 and DCAF13 in aged oocytes likely leads to the accumulation of MeCP2 protein, potentially offering insight into ovarian aging.

### Supplementary Information

Below is the link to the electronic supplementary material.

Supplementary file1 (PDF 1102 KB)

Supplementary file2 (XLSX 2638 KB)

Supplementary file3 (XLSX 2726 KB)

## Data Availability

The RNA-seq data generated in this manuscript will be deposited at GEO database. The RNA-seq data from human and mouse follicles were extracted from GEO database (GSE107746 and GSE135787) for RNA expression analysis for MeCP2 and DCAFs. The raw WGBS data reported in this paper have been deposited in the Genome Sequence Archive in National Genomics Data Center, China National Center for Bioinformation/Beijing Institute of Genomics, Chinese Academy of Sciences (GSA: CRA014212) that are publicly accessible at https://ngdc.cncb.ac.cn/gsa. The source data and any additional information in this paper will be shared upon reasonable request.
