# Supplementary material for: CRL4DCAF13 E3 ubiquitin ligase targets MeCP2 for degradation to prevent DNA hypermethylation and ensure normal transcription in growing oocytes
Source: Cell Mol Life Sci. 2024 Apr 5;81(1):165. doi: 10.1007/s00018-024-05185-4 (PMC10997554; doi:10.1007/s00018-024-05185-4)
Supplement: Supplementary file 1 — Supplementary file1 (PDF 1102 KB) [file 18_2024_5185_MOESM1_ESM.pdf]

Supplementary Figures

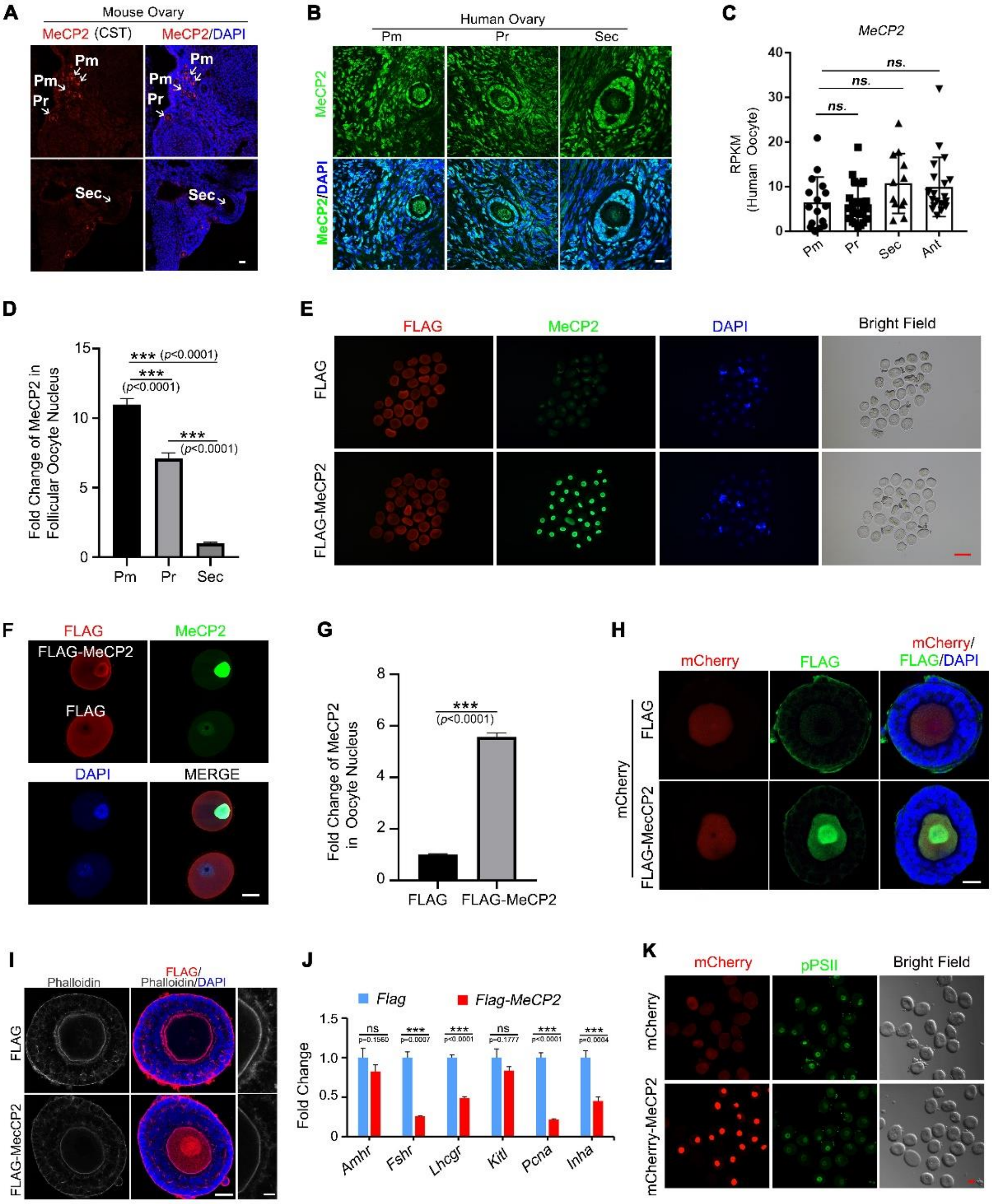

Supplementary Figure 1. The expression dynamics of MeCP2 and the role of MeCP2 overexpression on oocytes or ovarian follicle development.

A. Representative fluorescent images showing the MeCP2 expression pattern in wild-type mouse ovary using another anti-MeCP2 antibody. Pm, primordial follicles; Pr, primary follicles, Sec, secondary follicles. Scale bar = 10 μm.

B. Representative images of follicles at different stages from human ovary stained with anti-MeCP2 antibody (ab2828). Nuclei are labeled with DAPI (blue). Pm, primordial follicles; Pr, primary follicles, Sec, secondary follicles. Scale bar = 20 μm.

C. The mRNA expression dynamics of MeCP2 in oocytes from different stages of human follicles. Data were extracted from the GSE107746 database. RPKM, reads per kilobase per million mapped reads.

D. Fold change of MeCP2 in primordial and primary follicular oocytes relative to secondary. Pm, primordial follicles; Pr, primary follicles; Sec, secondary follicles. Data are expressed as mean ± SEM (n = 31-32). ***$P < 0.001$.

E-F. Immunostaining of MeCP2 using growing oocytes with or without *Flag-Mecp2* mRNAs. Scale bar = 100 μm (E) and 20μm (F).

G. Fold change of MeCP2 in growing oocytes with *Flag-Mecp2* mRNAs relative to *Flag*. Data are presented as means ±SEM (n = 32–33). Unpaired two-tailed Student's *t*-test. ***$P < 0.001$.

H. Immunostaining of mCherry-positive follicles after mRNAs microinjection with anti-FLAG antibody. Scale bar = 20 μm.

I. Immunofluorescence results for F-actin in control and oocyte-MeCP2 overexpression follicles. Scale bar = 20 μm, enlarged images scale bar = 5 μm.

J. The qRT-PCR results showing the relative levels of the indicated genes in granulosa cells derived from control and oocyte MeCP2-overexpressed follicles. Data are expressed as mean ± SEM (n = 3 technical replicates). ***$P < 0.001$.

K. Immunostaining of pPSII using growing oocytes with or without *mCherry-Mecp2* mRNAs. Scale bar = 20μm.

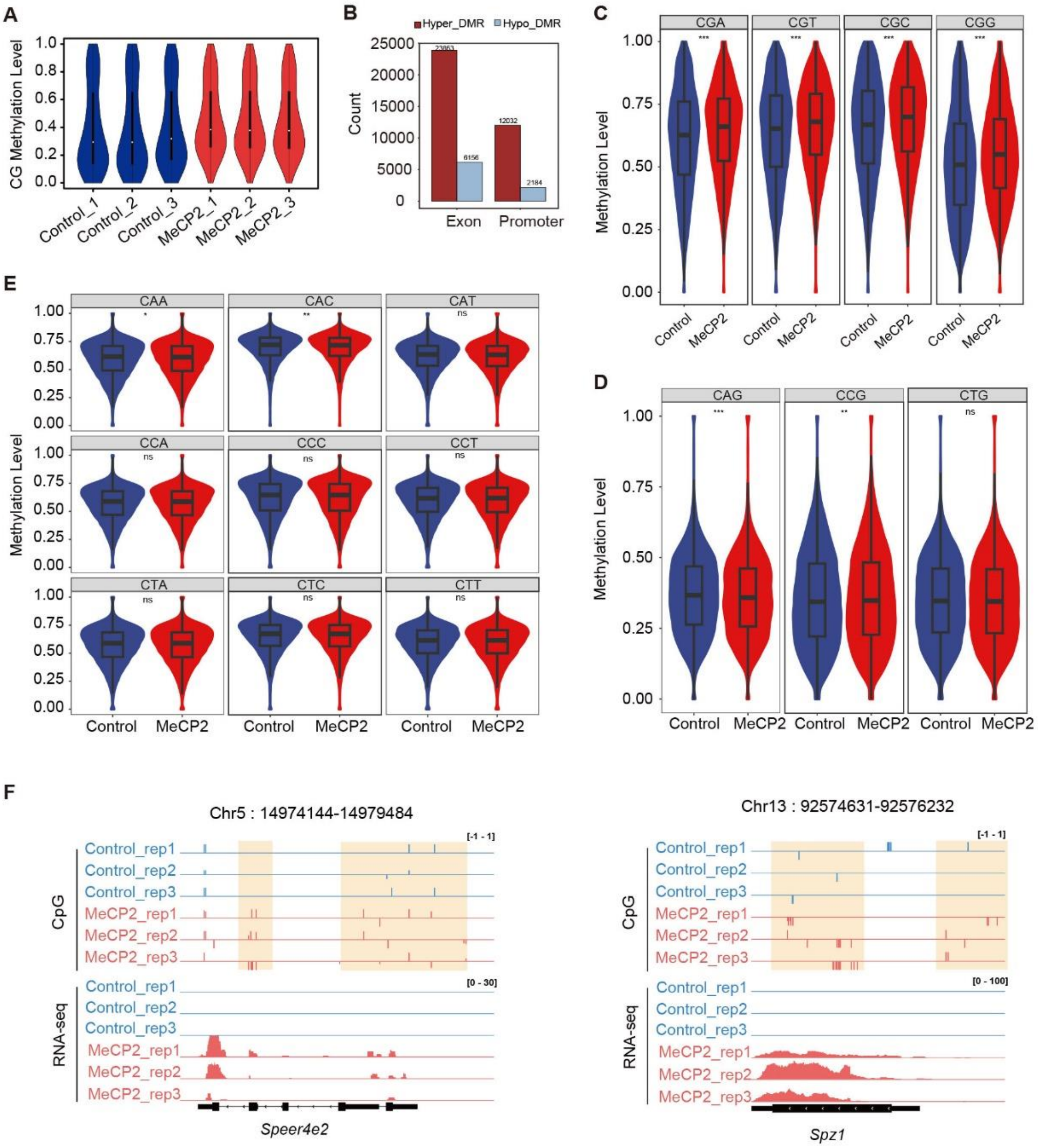

Supplementary Figure 2. The expression dynamics of different DCAFs in ovarian follicles.

A. Violin plots showing the DNA methylation level over 10-kb genomic bins in control and MeCP2 overexpression group.

B. The number of differential methylation regions (DMRs) distribution (including hyper-DMR and hypo-DMR) in exon and promoter regions in control and MeCP2-overexpressed oocytes.

C. Violin plots showing the methylation level of CG sub-contexts (CGA, CGT, CGC and CGG). ****P*<0.0001.

D-E. Violin plots showing the methylation level of CHG sub-contexts (D) and CHH sub-contexts (E). **P*<0.05. ***P*<0.001. ****P*<0.0001. ns, not significant.

F. The genome browser view of upregulated genes (*Speer4e* and *Spz1*) in RNA-seq

results with hyper-DMR in MeCP2-overexpression oocytes relative to control oocytes. DNA methylation levels of cytosines are indicated by the heights of the vertical bars on each track (CpG panel).

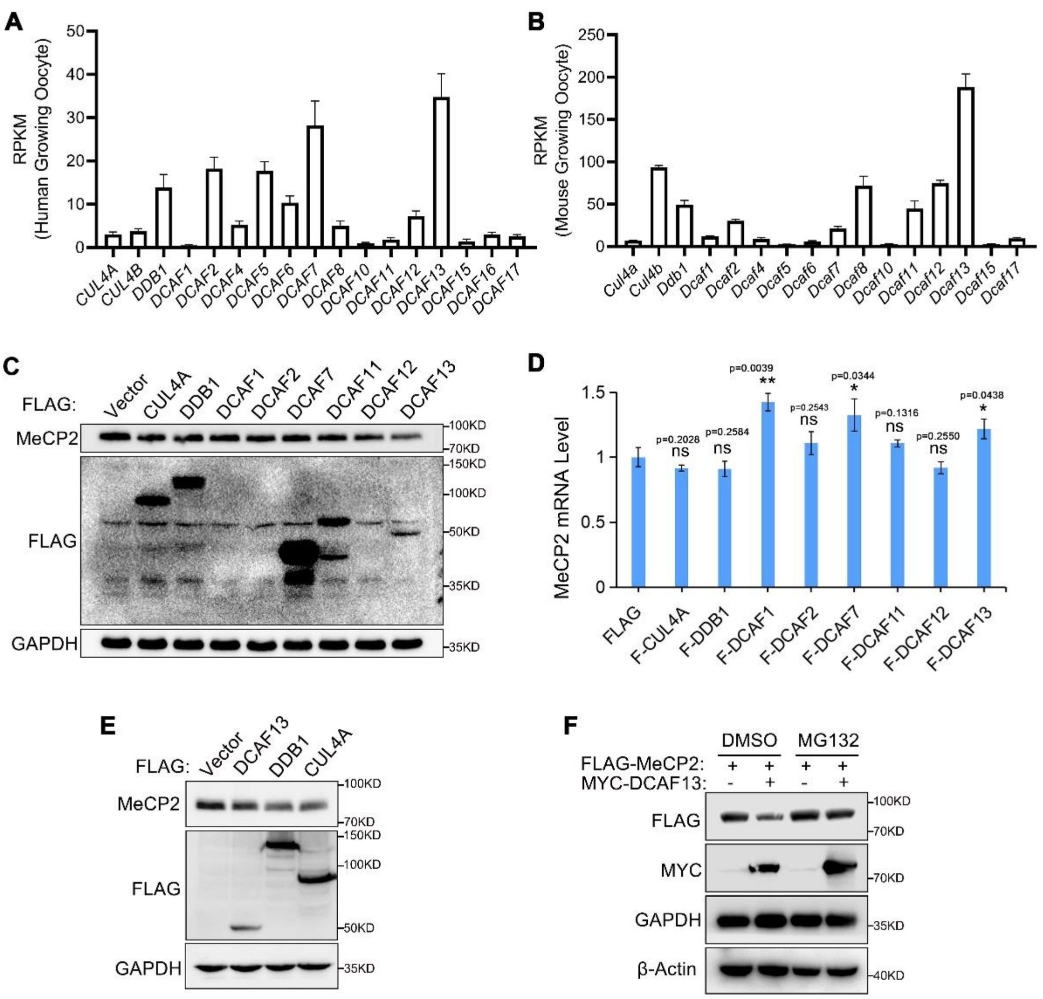

Supplementary Figure 3. The expression dynamics of different DCAFs in ovarian follicles.

A. The mRNA expression level of CUL4A/B, DDB1, and different DCAFs in human-growing oocytes. Data were extracted from the GSE107746 database. RPKM, reads per kilobase per million mapped reads.

B. The expression level of *Cul4A/B*, *Ddb1*, and different DCAFs in mouse-growing oocytes. Data were extracted from the GSE135787 database. RPKM, reads per kilobase per million mapped reads.

C. Western blotting results showing the MeCP2 and FLAG expression in HeLa cells transfected with indicated plasmids. GAPDH was used as the protein-loading control.

D. qRT–PCR displaying the relative levels of the *Mecp2* transcripts in Hela cells overexpressing indicated proteins. n = 3 technical replicates. Error bars, SEM (*P < 0.05; **P < 0.01).

E. Western blotting results showing the MeCP2 and FLAG expression in HeLa cells transfected with FLAG, FLAG-DCAF13, FLAG-DDB1 and FLAG-CUL4A plasmids. GAPDH was used as the protein-loading control.

F. Western blotting results demonstrating the FLAG and MYC expression in Hela cells transfected with indicated plasmids followed by MG132 disposing. GAPDH and β-Actin were blotted as the loading controls.

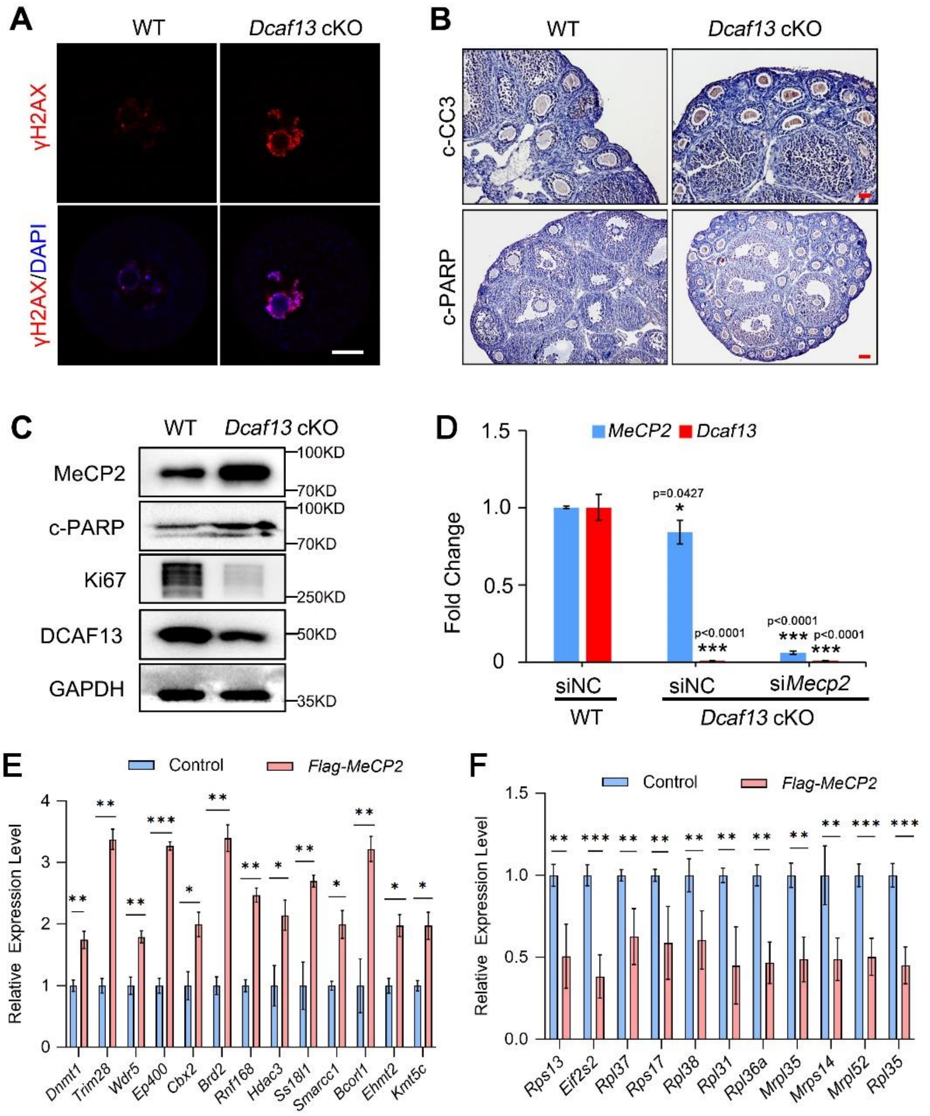

Supplementary Figure 4. The effect of deletion of *Dcaf13* on MeCP2, cell proliferation, and apoptosis.

A. Immunofluorescence results for γH2AX (red) in WT (*Dcaf13*$^{f/f}$) and *Dcaf13* cKO (*Dcaf13*$^{f/f}$*; Gdf9-Cre*) oocytes. The nuclei were indicated with DAPI (blue). Scale bar = 10 μm.

B. Immunohistochemistry (IHC) of cleaved caspase3 (c-CC3, upper panel) and cleaved PARP (c-PARP, lower panel) using ovaries from WT and *Dcaf13* cKO mice. Scale bar, upper panel = 50 μm, lower panel = 100 μm.

C. Western blotting results showing the MeCP2, C-PARP, Ki67, and DCAF13 expression in ovaries derived from WT and *Dcaf13* cKO mice. GAPDH was used as the protein-loading control.

D. qRT–PCR displaying the relative levels of the *Mecp2* and *Dcaf13* transcripts in

WT and *Dcaf13* cKO follicle oocytes injected with indicated siRNAs. n = 3 technical replicates. *$P < 0.05$; ***$P < 0.001$.

E-F. qRT–PCR results displaying the relative levels of the genes relative to covalent chromatin modification (E) and genes relative to translation (F) in growing oocytes with *Flag* (Control) or *Flag-MeCP2* overexpression. n = 3 technical replicates. *$P < 0.05$; **$P < 0.01$; ***$P < 0.001$.

Supplementary Tables

Supplementary Table 1. siRNA sequences

**Target**	**Sequences (5’-3’)**
NC	UUCUCCGAACGUGUCACGUTT
Dcaf13	GUGCUUACAUCACGAGAAATT
Ddb1	GGCCAAGAACAUCAGUGUGTT

Supplementary Table 2. Antibody information

**Protein name**	**Manufacture (catalogue number)**	**Applications (working dilution)**
MeCP2	Abcam ab253197	IF 1:500; WB 1:1000
GAPDH	ABclonal AC002	WB 1:1000
pPSII	Abcam ab5095	IF 1:600; WB 1:1000
FLAG	Sigma F1804	IF 1:200; WB 1:1000
ACTIN	Protech 60008	WB 1:1000
DDB1	Abcam ab109027	IF 1:100; WB 1:1000
DCAF13	Generated	WB 1:1000; IHC 1:100
MYC	CST 2278	WB 1:1000
HA	CST 3724	WB 1:1000
RDX	Abcam ab52495	IF 1:10; WB 1:1000
GDF9	Santa Cruz sc-12244	WB 1:100
BMP15	Abcam ab108413	WB 1:1000
FOXL2	Abcam ab5096	WB 1:500
H3	CST 4499	WB 1:1000
5mC	CST 28692	IF 1:1600
5hmC	Active Motif 39092	IF 1:100
MeCP2	CST 3456	IF 1:200
Ki67	Abcam ab15580	IF 1:600; WB 1:500
PCNA	Abcam ab18197	IF 1:200
C-PARP	CST 94885	WB 1:1000; IHC 1:100

pH2AX	CST 9718	IF 1:400
C-CC3	CST 9664	IHC 1:2000

Supplementary Table 3. Primer sequences

**Primer name**	**Sequences (5'-3')**	**Application**
c-Kit	F: TGCGTGTACACATTTGAAAGTG	Real-time PCR
	R: GAATGTTGGCCTTTTCAAGG	
Zar1	F: AGAGCGCCTATGTGTGGTGT	Real-time PCR
	R: TCTCCCACACAAGTCTTGCC	
Nobox	F: CATGAAGGGGACCTGAAGAA	Real-time PCR
	R: GGAAATCTCATGGCGTTTGT	
Oct4	F: CCGGAAGAGAAAGCGAACTA	Real-time PCR
	R: CTCATACTCTTCTCGTTGGGAAT	
Bmp15	F: TTATACCATCGTTCGGCTGAC	Real-time PCR
	R: CGGTAAACCACAGTGGCTCTGAT	
Fgf8	F: CTTTTGGAAGCAGAGTCCGA	Real-time PCR
	R: CCATGTACCAGCCCTCGTAC	
Cx37	F: CCCACATCCGATACTGGGTG	Real-time PCR
	R: CGAAGACGACCGTCCTCTG	
Bmp5	F: TTACTTAGGGGTATTGTGGGCT	Real-time PCR
	R: CCGTCTCTCATGGTTCCGTAG	
Gdg9	F: TCACCTCTACAATACCGTCCG	Real-time PCR
	R: CAGGTCACAGTGGAGGAGGA	
Actin	F: GCTCTTTTCCAGCCTTCCTT	Real-time PCR
	R: GTACTTGCGCTCAGGAGGAG	
Amhr2	F: GGGGCTTTGGACACTGCTT	Real-time PCR
	R: GTCTCGGCATCCTTGCATCTC	
Fshr	F: CCTTGCTCCTGGTCTCCTTG	Real-time PCR
	R: CTCGGTCACCTTGCTATCTTG	

Lhr	F: CGCCCGACTATCTCTCACCTA	Real-time PCR
	R: GACAGATTGAGGAGGTTGTCAAA	
Kitl	F: GAATCTCCGAAGAGGCCAGAA	Real-time PCR
	R: GCTGCAACAGGGGGTAACAT	
Pcna	F: TTTGAGGCACGCCTGATCC	Real-time PCR
	R: GGAGACGTGAGACGAGTCCAT	
Inhibin A	F: CCTTTTGCTGTTGACCCTACG	Real-time PCR
	R: AGGCATCTAGGAATAGAGCCTTC	
